# H3K18 lactylation-hexokinase 2 positive feedback loop promotes osteogenesis of ASPCs in facial infiltrating lipomatosis

**DOI:** 10.1186/s13287-025-04651-5

**Published:** 2025-10-01

**Authors:** Hongrui Chen, Chen Hua, Shih-Jen Chang, Yajing Qiu, Xiaoxi Lin, Bin Sun

**Affiliations:** Department of Plastic & Reconstructive Surgery , Shanghai Ninth People’s Hospital Shanghai Jiao Tong University School of Medicine , Shanghai, 200011 P.R. China

**Keywords:** Facial infiltrating lipomatosis, Adipose stem and progenitor cells, Osteogenesis, H3K18 lactylation, Hexokinase 2

## Abstract

**Background:**

Facial infiltrating lipomatosis (FIL) is a rare congenital disorder characterized by adipose hyperplasia and osseous overgrowth, driven by somatic PIK3CA mutations. While PIK3CA-induced metabolic reprogramming elevates lactate levels, the role of histone lactylation in FIL pathogenesis remains unclear.

**Methods:**

Adipose stem and progenitor cells (ASPCs) from FIL adipose tissue were isolated. Glycolysis inhibitors (2-DG, oxamate), lactate supplementation, and siRNA-mediated knockdown were used to modulate lactylation. CUT&Tag sequencing, Western blot, qPCR, ChIP-qPCR and functional assays (osteogenic/adipogenic differentiation) were performed to elucidate the potential mechanism.

**Results:**

FIL-ASPCs exhibited hyperlactylation, particularly at H3K18. H3K18la promoted osteogenesis by activating osteogenic genes, while adipogenesis remained unaffected. Inhibition of lactylation via glycolysis inhibitors or LDHA/LDHB knockdown suppressed osteogenic differentiation, whereas lactate supplementation reversed these effects. TGF-β1 stimulation could increase lactylation levels and promote osteogenic differentiation. Moreover, H3K18la upregulated hexokinase 2 (HK2), enhancing glycolysis and lactate production, thereby forming a lactate-H3K18la-HK2 positive feedback loop.

**Conclusions:**

This study identified H3K18 lactylation as a key epigenetic driver of FIL-associated osseous hyperplasia via a lactate-H3K18la-HK2 feedback loop. Targeting this axis may offer therapeutic potential for FIL and related metabolic bone disorders.

**Supplementary Information:**

The online version contains supplementary material available at 10.1186/s13287-025-04651-5.

## Introduction

Facial infiltrating lipomatosis (FIL) is a rare congenital disorder characterized by progressive facial enlargement evident at birth [1]. This enlargement arises primarily from aberrant subcutaneous adipose tissue hyperplasia and osseous overgrowth. FIL severely compromises maxillofacial function and aesthetics, profoundly affecting patients’ quality of life and mental health [2]. Somatic PIK3CA mutations identified in both bone and adipose tissues of FIL patients implicate this gene as a potential driver of the hyperplasia phenotypes [3].

Under aerobic conditions, owing to the “Warburg effect,” tumor cells tend to generate notably large amounts of lactic acid compared to normal cells, resulting in increased lactylation of proteins [4]. Histone lactylation, a novel epigenetic modification identified in 2019, occurs when lactate derived from glycolysis covalently modifies lysine residues on histones [5]. This process directly stimulates gene transcription by altering chromatin structure and recruiting transcriptional regulators [6]. Since its discovery, histone lactylation has been implicated in diverse pathological processes beyond cancer, including immune regulation, infection response, and aging-related diseases. For example, H3K14la directly upregulated p53 transcription and then contributed to pulmonary epithelial cell senescence during pulmonary fibrosis progression [7]. Another study found that glycolytic reprogramming in microglia and macrophages enhanced histone lactylation-mediated Cxcl16 transcription, thereby recruiting CD8^+^ T cells and further contributing to neuronal loss [8]. Additionally, H3K18la has been demonstrated to promote chondrogenesis and cartilage matrix deposition by enhancing the chromatin accessibility of chondrogenic genes [9]. Currently, research on the effects of histone lactylation on adipocytes remains limited.

Interestingly, previous study revealed that PIK3CA gain-of-function mutation in adipose tissue induced metabolic reprogramming with Warburg-like effect, promoting lactate accumulation in adipocytes [10]. However, the role of lactylation in the pathological process of FIL remains largely unknown. In this study, we isolated adipose stem and progenitor cells from FIL (FIL-ASPCs) and investigated the impact of lactylation on their function. Strikingly, we found that H3K18 lactylation (H3K18la) promoted osteogenic differentiation of FIL-ASPCs but not adipogenesis. Mechanistically, cleavage under targets and tagmentation (CUT&Tag) analysis revealed that H3K18la enhanced the transcription of a subset of osteogenesis-related genes. Furthermore, H3K18la upregulated hexokinase 2 (HK2) expression, further exacerbating glycolysis and lactate accumulation, thereby establishing a lactate-H3K18la-HK2 positive feedback loop. Our findings provide novel insights into the mechanisms underlying the bony hyperplasia deformities observed in FIL.

## Methods

The work has been reported in line with the ARRIVE guidelines 2.0.

### Human subject and ethics approval

The patients’ clinical characteristics were presented in Table S1. Adipose tissues from FIL patients and patients underwent facial cutaneous lesions surgery (CON) were collected for experimental analyses. This study was conducted in accordance with the Declaration of Helsinki and approved by the Ethics Committee of Shanghai Ninth People’s Hospital. The approval number was No.SH9H-2022-T215-1. Date that the ethics approval was granted: October 6, 2022. ASPCs were isolated and collected from facial adipose tissue, with written consent of donors.

### Cell culture

ASPCs were isolated from adipose tissue and identified as described previously [11]. Cells were cultured in basal growth medium (Cyagen, HUXMD-90011) containing 10% fetal bovine serum, 1% penicillin and streptomycin, and maintained in a humidified atmosphere containing 5% CO2 at 37 °C. ASPCs were digested with 0.25% trypsin (Gibco, 25200056) and passaged routinely when 80–90% confluence was reached. ASPCs from passages 3–6 were used in the following experiments.

### Western blot

Lysates from ASPCs cells or tissues were prepared using RIPA buffer (Epizyme, PC101) supplemented with 1% phosphatase inhibitor mix (Epizyme, GRF102) and 1% protease inhibitor blend (Epizyme, GRF101) at a temperature of 4 °C for a duration of 20 min. Protein concentrations were determined by the bicinchoninic acid assay (Epizyme, ZJ102), and samples were adjusted to equalize their protein content. The proteins were combined with a protein sample buffer (Epizyme, LT101) in a ratio of 4:1, and then subjected to a heat treatment at 100 °C for 7 min prior to being resolved on 0.45 μm PVDF membranes. After a 15-minute blocking step with a Protein Free Rapid Blocking Buffer (1×) (Epizyme, PS108P), the membrane was incubated overnight with primary antibodies, including pan anti-kla (PTM Bio, PTM-1401RM), anti-H3K18la (PTM Bio, PTM-1427RM), anti-H3K9la (PTM Bio, PTM-1419RM), anti-H3K23la (PTM Bio, PTM-1413RM), anti-H4K5la (PTM Bio, PTM-1407RM), anti-H4K12la (PTM Bio, PTM-1411RM), anti-α tubulin (Proteintech, 11224-1-AP), anti-HK2 (Epizyme, R010880), anti-LDHA (Cell Signaling Technology, 2012), anti-Histone H3 (HUABIO, M1309-1), anti-Histone H4 (HUABIO, ET1612-43), anti-LDHB (Cell Signaling Technology, 56298), anti-RUNX2 (Proteintech, 20700-1-AP), anti-Collagen type I (COL-1, Proteintech, 67288-1-Ig), anti-ALP (Abcam, ab307726), anti-PPAR γ (Cell Signaling Technology, 2443 S), anti-C/EBP α (Proteintech, 13274-1-AP), and anti-FABP 4 (Proteintech, 12802-1-AP) overnight. Subsequently, the membranes were incubated with secondary antibodies for 45 min at room temperature and visualized using NcmECL Ultra (NCM Biotech, Suzhou, China) with the Tanon 4600 Automatic Chemiluminescence/Fluorescence Image Analysis System (Tanon, Shanghai, China). The intensity of the immunoreactive bands was quantified using Image J software. All uncropped blot can be found in Figure S5.

### Oil red O staining

Oil Red O staining was performed according to the instructions of the Modified Oil Red O Staining Kit (Beyotime, C0158S). Quantitative analysis of Oil red O staining area was determined using Image-Pro Plus 6.0 software.

### Osteogenic induction

Once the cells reached confluency, the growth medium was switched to osteogenic differentiation kit (Cyagen Biosciences, HUXMD-90021) for 5 or 9 days. Induction solution was mixed with various concentrations of 2-Deoxy-D-glucose (2-DG, MedChemExpress, HY-13966), Oxamic acid sodium (Oxamate, MedChemExpress, HY-W013032A), sodium lactate (Nala, MedChemExpress, HY-B2227B) when needed.

### Adipogenic induction

Once the cells reached confluency, the growth medium was switched to induction medium A. After three days, the medium was replaced by induction medium B for one day (all from Cyagen Biosciences, USA). Induction A and B were mixed with various concentrations of 2-DG, Oxamate, and Nala when needed. The cells were used on days 8 for follow-up experimental research.

### Alizarin red staining (ARS)

ARS was performed according to the instructions of the Alizarin Red Staining Solution (Beyotime, C0138). Quantitative analysis of ARS area was determined using Image-Pro Plus 6.0 software.

### Alkaline phosphatase (ALP) staining

ALP staining was performed after osteogenic induction according to the manufacturer’s instruction (Abcam, ab284936).

### Cell counting Kit-8 (CCK-8) assay

Herein, 8 × 10^3^ cells/well were inoculated in a 96-well plate. After complete attachment of the cells, drugs were added, and appropriate drug concentration gradients were set. The cell activity was measured after 48 h using a CCK-8 kit (Yeasen, 40203ES60), and the corresponding cellular viability were calculated.

### Lactic acid content detection

Digest the cells in a 10 cm culture dish and take 2 × 10^7^cells for detection. Perform the experiment according to the instructions of the reagent manufacturer (Lactic acid content (LA) detection kit, Solarbio), use an Enzyme labeled instrument to detect the absorbance value at 570 nm, and calculate the corresponding lactic acid concentration.

### Immunofluorescence

FIL adipose tissues and normal adipose tissues in paraffin-embedded sections were deparaffinized, rehydrated, fixed, and blocked with 5% normal goat serum (Vector) and then incubated with pan anti-Kla (PTM-1401RM) and anti-H3K18la (PTM-1427RM) antibodies at 4 °C overnight. Thereafter, the slides were incubated with the appropriate secondary antibodies for 30 min, and nuclei were counterstained with DAPI (Sigma-Aldrich) for 1 h. Digital images were obtained with a ZEISS Axio Scope A1 upright microscope. Relative pan-Kla levels and H3K18la levels were determined by comparing the fluorescence intensity of the target antibody with that of DAPI.

### Histological staining

After CT analysis, the formation of new bone tissue at the defect site was further examined by histological staining. Briefly, femoral specimens were fixed with 4% paraformaldehyde for 48 h. Subsequently, they were continuously decalcified with phosphate containing 12.5% ethylenediaminetetraacetic acid (EDTA) disodium salt, and the solution was replaced with fresh solution every 48 h. After decalcification, they were embedded in paraffin and routinely sectioned for H&E, Masson, toluidine blue and Saf-O/Fast Green staining.

### Methods-Cleavage under targets and tagmentation (CUT&Tag) assays

The CUT&Tag assay was conducted using the Hyperactive™ In-Situ ChIP Library Prep Kit for Illumina(Vazyme Biotech, China) in accordance with the manufacturer’s instructions. After being washed with PBS,cultured primary ASPCs were incubated with ConA Beads, primary antibody against H3K18la (PTM Bio,PTM-1427RM), secondary antibody and the Hyperactive pA-Tn5 Transposase in order and then fragmented.The fragmented DNA was then isolated, amplified, and purified for sequencing and qPCR assays. ForCUT&Tag sequencing, the libraries were sequenced on an Illumina NovaSeq6000 platform (GenefundBiotech, Shanghai, China). The clean reads were obtained after removing adapter sequences and low-qualityreads with Fastp software, and mapped to the human reference genome using Bowtie2. Peak calling wasperformed with SEACR software and annotated using CHIPseeker software. IGV software was used tovisualize peak distributions along genomic regions of interested genes.

### Quantitative real-time PCR (qPCR)

Total RNA was isolated using Trizol reagent (Thermo Scientific). Then, RNA was reverse-transcribed into cDNA using a 1 st Strand cDNA synthesis kit (Yeasen, 11119ES60). qPCR was performed using SYBR green mix (Vazyme) on an Applied Biosystems (Thermo Scientific). Relative mRNA expression was calculated using the 2^−ΔΔCt^ method as normalized to GAPDH. The primers are shown in Table S2.

### Chromatin Immunoprecipitation followed by qPCR (ChIP-qPCR)

ChIP was performed according to the manufacturer’s instructions (The Agarose ChIP Kit, 26 156, ThermoFisher). Summarily, FIL-ASPCs were fixed with 1% formaldehyde for 10 min at room temperature and quenched with Glycine Solution (1X) for 5 min. Then, fixed cells were lysed with Lysis Buffer 1 containing protease inhibitors and digested with Micrococcal Nuclease (ChIP Grade) (10 U/µL). The digested chromatin was incubated Dilution Buffer containing primary antibodies at 4 °C overnight on a vertical roller. Then, ChIP Grade Protein A/G Plus Agarose was added to each IP, the column was centrifuged, and the column was washed with Wash Buffer 1/2/3. Later, IP was eluted with 150 µL IP Elution Buffer, 6 µL of 5 m NaCl, and 2 µL of 20 mg mL^−1^ Proteinase K. Finally, DNA was obtained by DNA Binding Buffer, DNA Clean-Up Column, and DNA Column Wash Buffer following the protocols. Purified DNA levels were quantitatively measured by real-time PCR.

### Lentivirus packaging

We prepared lentivirus carrying shRNA to knock down the expression of HK2. The lentivirus was obtained from Zuorun Biotech. For transduction, ASPCs were incubated with virus-containing supernatant (MOI = 10) in the presence of 10 µL polybrene. After 10 h, infected cells were selected with puromycin supernatant.

### Plasmid transfection

Three siRNA sequences targeting LDHA and LDHB were designed, with the most effective siRNA sequence used for subsequent transfections. The siRNA sequences were designed and synthesized by GenePharma (Shanghai, China). Transfections were carried out according to the instructions of Lipofectamine 8000 (Beyotime, C0533).

### Animal models

A total of 10 male adult SD rats were randomly divided into two groups (*n* = 5 per group): control (group 1) and siLDHA/LDHB (group 2) groups. The rats were anesthetized by injecting sodium pentobarbital intraperitoneally. Then, 4 cm longitudinal skin incision was made along the femur to separate the musculature of the lateral femur. A defect with a diameter of 3 mm and a depth of 3 mm was created using a depth-limiting drill. Matrigel mixed with human FIL-ASPCs was subsequently implanted into the defects. After rewashing the wound with iodophor and saline, the muscle and skin were sutured. Antibiotics were intramuscularly administered for 5 consecutive days after operation. All rats receiving human FIL-ASPCs implantation were administered cyclosporine A (CsA, 15 mg/kg/day) via oral gavage, initiated 5 days preoperatively and continued daily until sacrifice. CsA was dissolved in olive oil (10 mg/mL) for precise dosing. The rats were executed by CO_2_ asphyxiation at 4 weeks postoperatively, and the intact femoral specimens were collected for further analysis.

### Quantification and statistical analysis

GraphPad Prism (v 8.0) was used for statistical analyses. Two-tailed Student’s t test was utilized for comparison between the two groups. One-way analysis of variance (ANOVA) coupled with the Bonferroni’s post hoc test was used for comparisons among three or more groups. Data were presented as mean ± standard deviation (SD). *P* < 0.05 was considered of statistical significance.

## Results

### Elevated histone lactylation levels in the adipose tissue and ASPCs of FIL

**Fig. 1:**
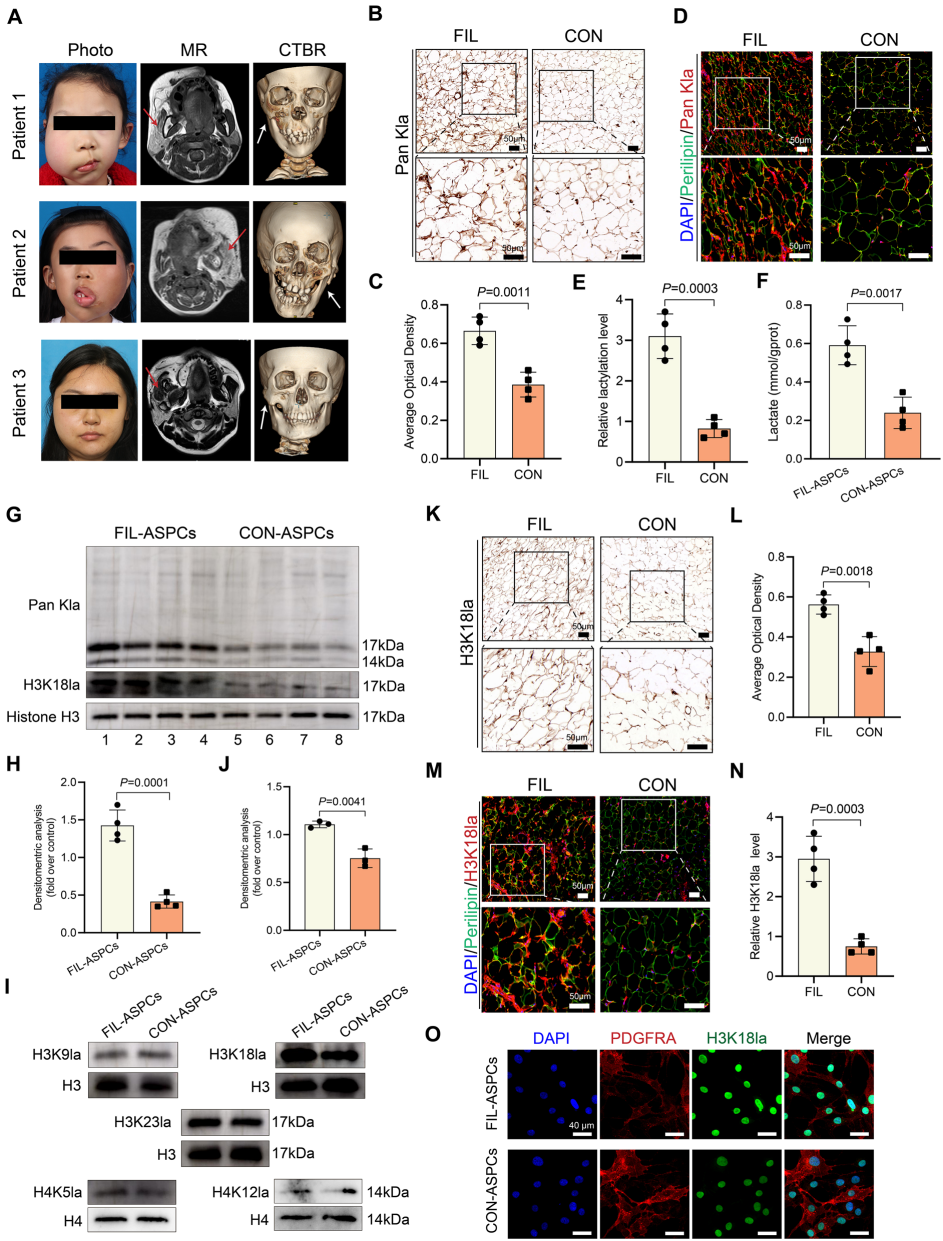
Increased lactylation level in FIL adipose tissue and FIL-ASPCs. **A** clinical photos and imaging images of FIL patients. **B** Immunohistochemical staining of the pan-kla in adipose tissue. **C** Quantitative analysis of immunohistochemical staining of pan-kla. **D** Lactylation levels were visualized by immunofluorescence staining in FIL and CON adipose tissue. **E** Statistical results of lactyation levels in FIL and CON adipose tissue. **F** Lactate level in FIL and CON adipose tissue. **G**, **H** Lactyation and H3K18la levels were detected FIL-ASPCs and CON-ASPCs by Western blot. I: Western blotting analysis of site-specific histone lactylation in FIL-ASPCs and CON-ASPCs. **J** Quantitative analysis of H3K18la level in FIL-ASPCs and CON-ASPCs. **K**, **L** Immunohistochemical staining and the quantitative analysis of the H3K18la in adipose tissue. **M** H3K18la levels were visualized by immunofluorescence staining in FIL and CON adipose tissue. **N** Statistical results of H3K18la levels in FIL and CON adipose tissue. **O** H3K18la levels were visualized by immunofluorescence staining in FIL-ASPCs and CON-ASPCs. Adipose tissue isolated from FIL patients No. 1–4 and CON patients No. 14–17 was used in immunohistochemical staining and immunofluorescence staining. ASPCs isolated from FIL patients No. 1–4 and CON patients No. 14–17 were used for Western blotting. Experiments were independently replicated at least three times with similar results (biological replicates). Data were analyzed by unpaired two-sided Student’s t tests (**C**, **E**, **F**, **H**, **J**, **L**, **N**) and were presented as mean ± SD with three replicate experiments. Full-length blots were presented in Figure S5

Photograph assessment confirmed substantial facial overgrowth in all FIL cases, where diagnostic imaging demonstrated coexisting osseous malformations and pathological adipose proliferation (Fig. 1A). Building upon our previous discovery of PIK3CA mutations in FIL-ASPCs and adipose specimens [11], and considering the established association between PIK3CA hyperactivity and Warburg effect-mediated lactate overproduction-a potential precursor for epigenetic lactylation [10], we conducted comparative lactylation profiling. Immunohistochemical and immunofluorescent evaluations revealed significantly elevated global protein lactylation in FIL adipose sections compared to healthy controls (Fig. 1B-E). Subsequently, we isolated ASPCs using enzymatic digestion and assessed their purity through flow cytometry analysis (Figure S1A). Metabolic characterization of cultured ASPCs demonstrated higher intracellular lactate concentrations in FIL-ASPCs versus CON-ASPCs (Fig. 1F). Immunoblot analyses confirmed this hyperlactylation phenotype in FIL-ASPCs, showing particularly intense lactylation signals at 17 kDa-a molecular weight corresponding to histone H3 (Fig. 1G-H). Targeted lactylation mapping identified H3K18la as the most pronounced modification site in FIL-ASPC lysates compared to controls (Fig. 1I-J). Consistent with cellular findings, adipose tissue analyses showed parallel increases in H3K18la signals across FIL specimens (Fig. 1K-N), with protein assessments confirming elevated H3K18la levels in FIL-ASPCs (Fig. 1G, O). These data collectively identified H3K18 hyperlactylation as a characteristic epigenetic alteration in FIL pathogenesis, potentially linking metabolic reprogramming to disease progression.

### Inhibition of H3K18la suppressed osteogenesis of FIL-ASPCs in vitro

**Fig. 2:**
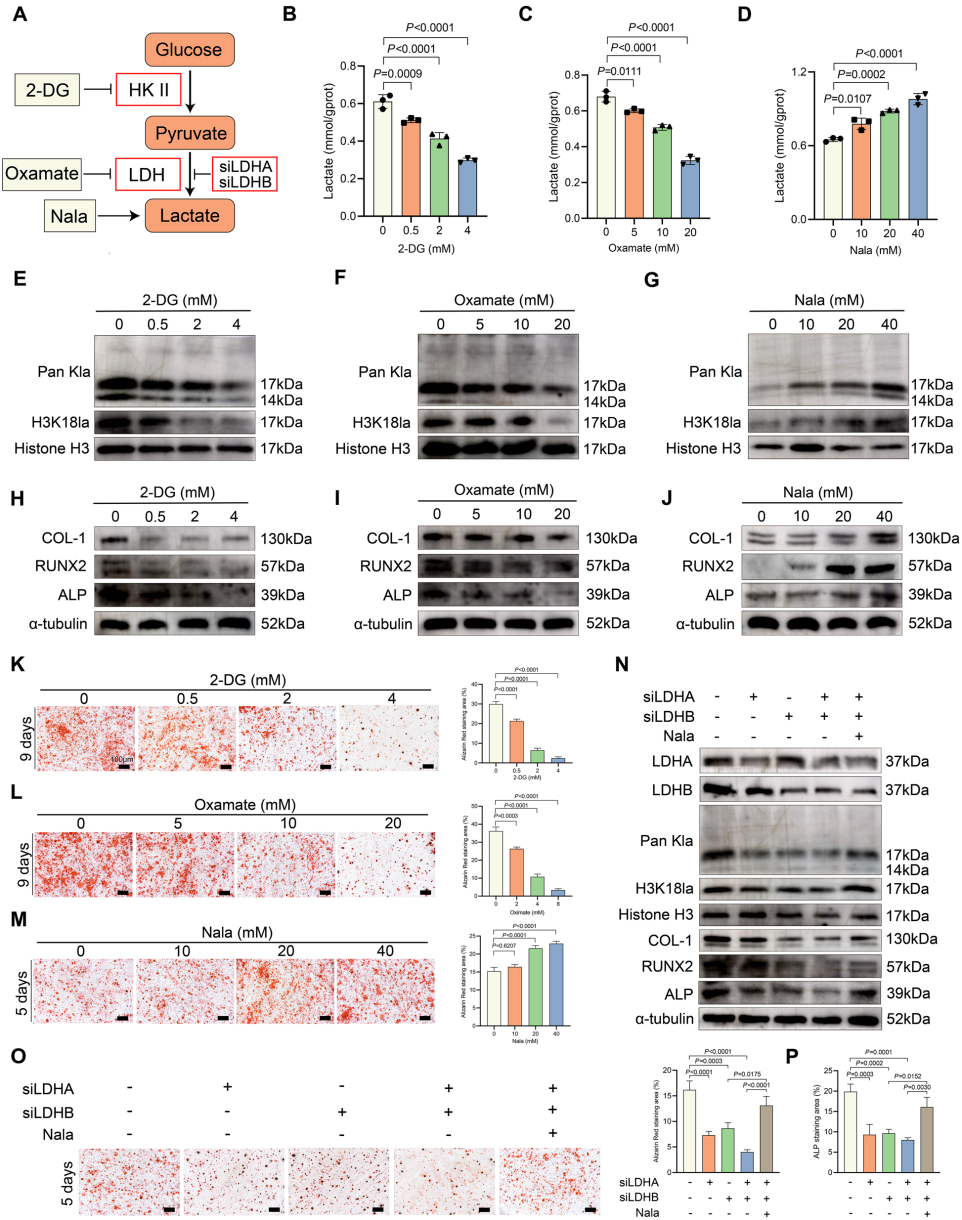
Inhibition of H3K18la inhibited osteogenesis in vitro. **A** Schematic diagram of histone lactylation inhibition methods target. **B**–**D** Intracellular lactate levels were measured from FIL-ASPCs cultured in different concentrations of 2-DG (**B**), oxamate (**C**) or Nala (**D**) for 24 h. **E**–**G** Lactyation and H3K18la levels were detected in FIL-ASPCs cultured in different concentrations of 2-DG (**E**), oxamate (**F**), and Nala (**G**) for 24 h by Western blot. **H**–**J** COL-1, RUNX2, ALP levels were detected in FIL-ASPCs cultured in osteogenic solution with different concentrations of 2-DG (**H**), oxamate (**I**), and Nala (**J**) for 3 days by Western blot. K-M: ARS staining of the FIL-ASPCs treated with different concentrations of 2-DG (**K**), oxamate (**L**), and Nala (**M**). N: Lactyation, H3K18la, COL-1, RUNX2, and ALP levels were detected in FIL-ASPCs treated with osteogenesis induction medium after LDHA and LDHB silencing by Western blot. **O** ARS staining of the FIL-ASPCs treated osteogenesis induction medium after LDHA and LDHB silencing and Nala supplement. **P** Quantitative analysis of ALP staining of FIL-ASPCs treated osteogenesis induction medium after LDHA and LDHB silencing. ASPCs isolated from FIL patients No. 1–3 and CON patients No. 14–16 were used for Western blotting and osteogenic induction. Data were analyzed by one-way ANOVA (**B**, **C**, **D**, **K**, **L**, **M**, **O**, **P**) and were presented as mean ± SD with three replicate experiments (biological replicates). Full-length blots were presented in Figure S5

To investigate histone lactylation’s regulatory role in FIL-ASPC adipogenesis, we implemented three metabolic interventions to regulate intracellular lactylation: (1) pharmacological glycolysis suppression using 2-DG and oxamate; (2) exogenous lactate elevation via Nala; (3) LDHA/LDHB siRNA-mediated lactate dehydrogenase silencing (Fig. 2A) [5]. Metabolic profiling demonstrated dose-responsive reductions in pan-histone lactylation and H3K18la signals following glycolysis inhibition, correlating with diminished intracellular lactate concentrations (Figs. 2B, C, E, F). Conversely, Nala supplementation elevated both lactate levels and lactylation markers (Figs. 2D, G). Subsequently, we induced adipogenic differentiation in FIL-ASPCs. Surprisingly, Oil Red O staining demonstrated that while 2-DG treatment markedly inhibited adipogenic differentiation, neither oxamate nor Nala significantly affected lipid accumulation during adipogenesis (Figure S2A, C, E). Western blot analysis further revealed that 2-DG treatment suppressed the expression of key adipogenic proteins (PPARγ, C/EBPα, and FABP4), whereas oxamate and Nala treatments showed no significant effects on their expression (Figure S2B, D, F). This discrepancy may be attributed to the fact that 2-DG broadly inhibited glycolysis by competitively blocking glucose metabolism. This not only reduced lactate production but also depleted downstream metabolites (e.g., acetyl-CoA, ATP) essential for adipogenesis [12].

Previous studies reported that endothelial cell-derived lactate promoted osteogenic differentiation of bone marrow mesenchymal stem cells (BMSCs), and LDHA knockdown impairs osteogenic differentiation in MC3T3-E1 mouse osteoblast precursor cells [13, 14]. Therefore, we next sought to investigate the role of H3K18la regulation in the osteogenic differentiation of FIL-ASPCs. We found that reduced histone lactylation efficiently suppressed the expression of key osteogenic factors (RUNX2) and osteogenic markers (COL-1 and ALP) in FIL-ASPCs (Fig. 2H, I). Conversely, Nala supplementation elevated the levels of these proteins (Fig. 2J). ARS demonstrated that 2-DG and oxamate significantly reduced mineralized nodule formation during osteogenic differentiation, whereas Nala accelerated this process (Fig. 2K**–**M). Since 2-DG and oxamate may exert off-target effects independent of lactate production and lactylation inhibition, we further silenced LDHA and LDHB to specifically inhibit histone lactylation. Notably, LDHA-deficient or LDHB-deficient FIL-ASPCs (Fig. 2N) exhibited a marked reduction in global histone lactylation. Moreover, simultaneous silencing of LDHA and LDHB significantly impaired histone lactylation and concurrently suppressed the expression of COL-1, RUNX2, and ALP. Additionally, reintroducing Nala into LDHA/LDHB-deficient cells restored histone lactylation levels and partially rescued COL-1, RUNX2, and ALP expression (Fig. 2N). ARS staining further revealed that knockdown of LDHA or LDHB in FIL-ASPCs under osteogenic induction reduced calcium deposition, and Nala supplementation partially reversed this effect (Fig. 2O). ALP staining showed similar results (Fig. 2P). Taken together, the regulation of histone lactylation exerted no significant effect on the adipogenic differentiation of FIL-ASPCs. However, inhibition of histone lactylation markedly impaired osteogenic differentiation, whereas supplementation with Nala to elevate histone lactylation levels promoted osteogenic differentiation in FIL-ASPCs.

### Inhibition of histone lactylation suppressed osteogenesis of FIL-ASPCs in vivo

**Fig. 3:**
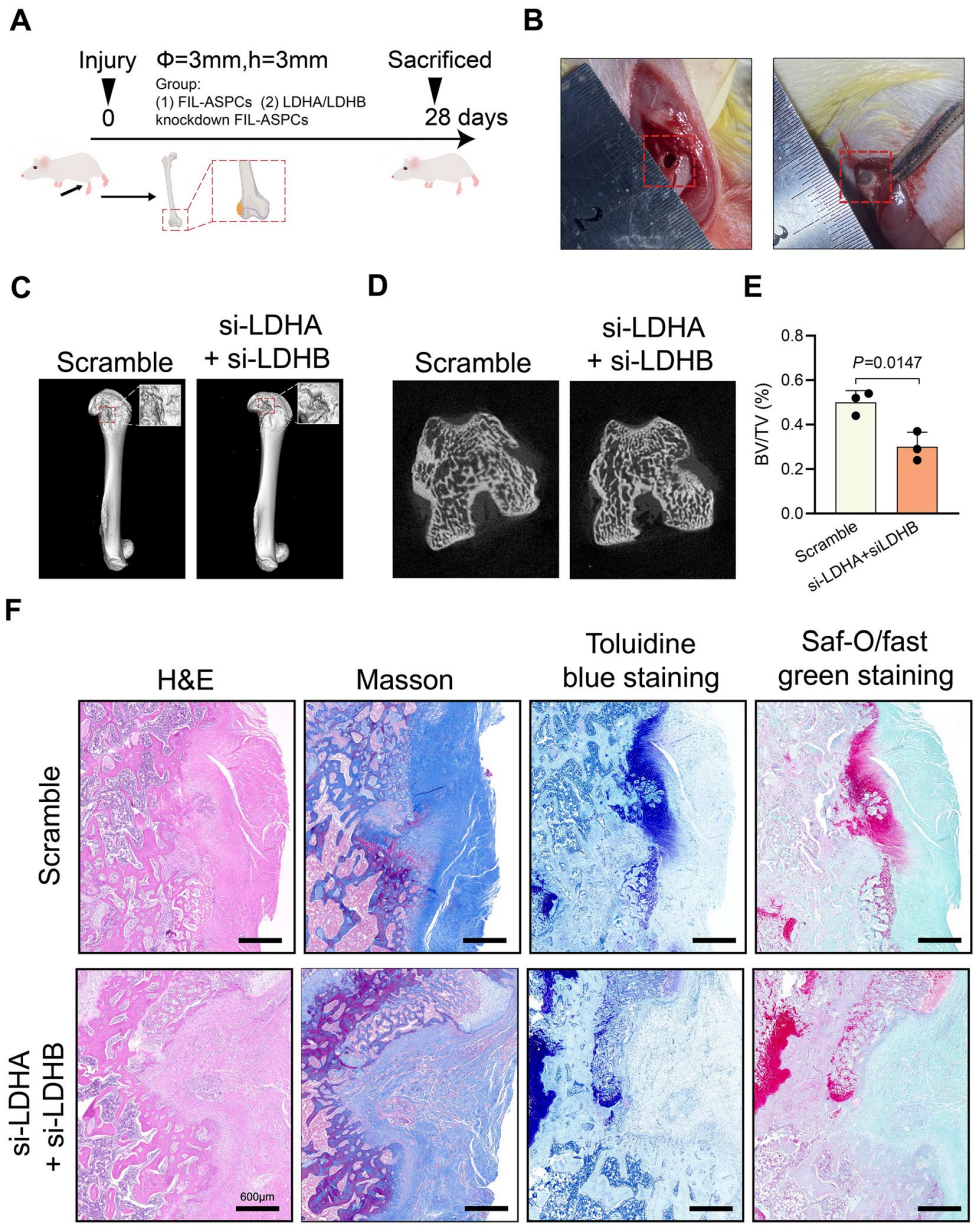
Osteogenesis and new bone formation were observed 28 days after surgery. **A** The overall flow chart of animal experiments and the schematic diagram of experimental groups. **B** The matrix gel was injected after the local bone defect was constructed. **C**, **D** Micro-CT reconstruction images of femur defect area 4 weeks after surgery. **E** bone volume to total volume (BV/TV) value was quantified using micro-CT in the region of interest. **F** H&E staining, Masson staining, toluidine blue staining, and Saf-O/fast green staining under light microscopy. Scale bar = 600 μm. ASPCs isolated from FIL patients No. 1–3 and CON patients No. 14–16 were used for in vivo experiments

While in vitro studies demonstrated impaired osteogenic differentiation in LDHA/LDHB-suppressed FIL-ASPCs, we investigated H3K18la’s bone regenerative potential through in vivo femoral defect models in rodents (Fig. 3A). Surgical implantation involved local administration of Matrigel-embedded FIL-ASPCs into osteotomy sites (Fig. 3B). Two experimental groups were established: one with FIL-ASPCs transfected with an empty plasmid (group 1) and another with FIL-ASPCs subjected to LDHA/LDHB knockdown (group 2). Quantitative bone restoration was assessed via micro-CT volumetric reconstruction, focusing on defect-adjacent regions within a 40 μm boundary. Postoperative imaging at 28 days revealed striking differences: control cohorts displayed extensive mineralization with dense trabecular networks, while LDH-deficient groups showed persistent cavity formation with sparse ossification (Figs. 3C-D). Additionally, the analysis of bone microstructure within the ROI indicated a reduced bone volume fraction (BV/TV) in the group receiving FIL-ASPCs with LDHA/LDHB knockdown (Fig. 3E).

To offer a comprehensive assessment of the newborn bone conditions beyond CT scans, we employed a diverse range of histological staining techniques, including H&E, Masson, toluidine blue, and Saf-O/Fast Green. The H&E staining highlighted a significant increase in new bone formation around the Matrigel in group (1) After Masson staining, group 1 showed superior osseointegration, with a larger amount of mature bone tissue (colored red) and a greater quantity of newly formed bone (colored blue) compared to group (2) Toluidine blue staining indicated a higher number of trabecular structures in group 1. Furthermore, Saf-O/Fast Green staining revealed a higher degree of bone formation (colored green) and cartilage formation (colored red) in group 1 (Fig. 3F).

### Identifcation of potential downstream targets of H3K18 lactylation by genome-wide CUT & Tag analysis

**Fig. 4:**
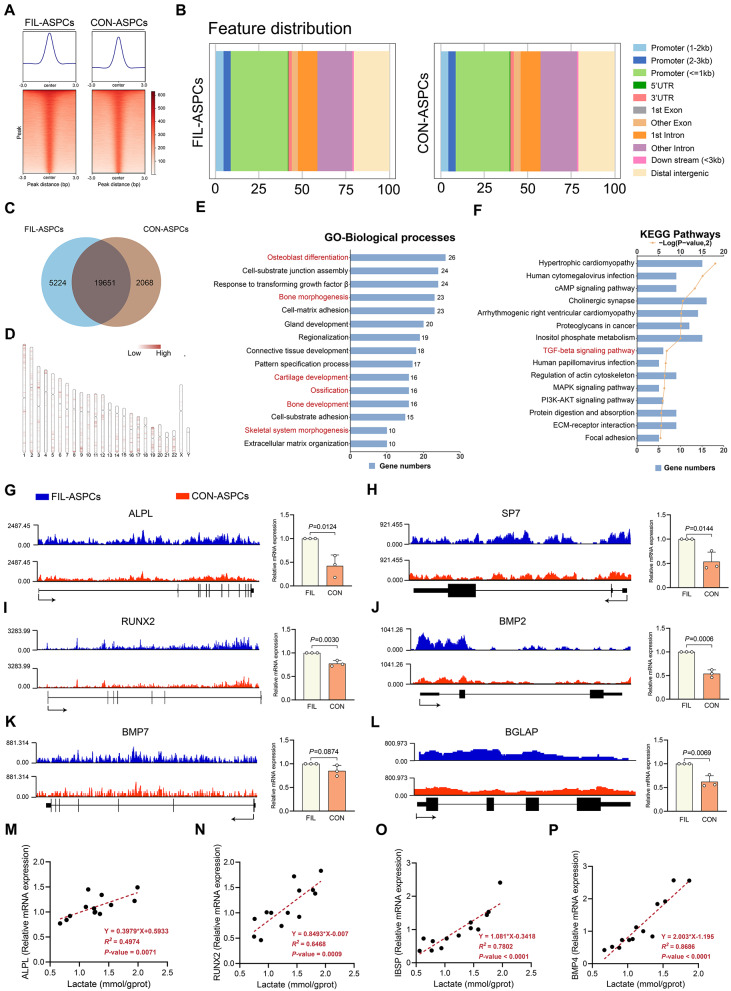
Identification of potential downstream targets of H3K18 lactylation by genome-wide CUT& Tag analysis. **A** The binding density of H3K18la was visualized by deepTools: the heatmap presents the CUT&Tag tag counts on the different H3K18la binding peaks in FIL-ASPCs and CON-ASPCs. **B** Genome-wide distribution of H3K18la enrichment peaks in FIL-ASPCs and CON-ASPCs. **C** The number of peaks identifed in FIL-ASPCs and CON-ASPCs. **D** Chromatin distribution of the H3K18la enrichment peaks in ASPCs. **E** GO analysis of the top 15 elevated H3K18a binding peaks at candidate target genes in FIL-ASPCs. **F** KEGG analysis of the top 15 elevated H3K18a binding peaks at candidate target genes in FIL-ASPCs. **G**–**L** Genome browser tracks of CUT& Tag signal at the ALPL (**G**), SP7 (**H**), RUNX2 (**I**), BMP2 (**J**), BMP7 (**K**) and BGLAP (**L**) loci and qPCR assays monitoring expression of ALPL (**G**), SP7 (**H**), RUNX2 (**I**), BMP2 (**J**), BMP7 (**K**) and BGLAP (**L**) in FIL-ASPCs and CON-ASPCs treated osteogenic induction medium. **M**–**P** Correlation analysis of ALPL (M), RUNX2 (**N**), IBSP (**O**), BMP4 (**P**) mRNA and lactate level. ASPCs isolated from FIL patients No. 1–3 and CON patients No. 14–16 were used for CUT & Tag analysis

We employed CUT&Tag, an ultra-sensitive chromatin profiling technique, to delineate H3K18la-mediated transcriptional regulation in ASPCs. Genome-wide epigenetic mapping compared H3K18la-associated genomic loci between FIL-ASPCs and control counterparts. DeepTools processing revealed pronounced chromatin occupancy of H3K18la in pathological ASPCs versus controls (Fig. 4A). Cross-group analysis identified 19,651 H3K18la binding peaks in both groups and over 35% of the binding peaks were located in promoter sequences (< 3 kb) (Fig. 4B-C). Interestingly, the genes regulated by H3K18la were highly clustered in certain chromosomal regions (Fig. 4D). To evaluate the epigenetic modulatory influence of H3K18la in ASPCs, the downstream genes with differential binding peaks were classified by gene ontology (GO) and Kyoto Encyclopedia of Genes and Genomes (KEGG) analyses. Interestingly, we found that the upregulated peak-related genes were enriched in several pathways related to osteogenesis, including osteoblast differentiation, bone morphogenesis, ossification, and cartilage development (Fig. 4E, F).

The pronounced enrichment of H3K18la at osteogenic gene loci in FIL-ASPCs prompted investigation into its functional correlation with skeletal differentiation markers. Epigenetic profiling via CUT&Tag revealed heightened H3K18la enrichment at promoters of osteogenesis-associated genes (ALPL, RUNX2, BMP7, SP7, BMP2, BGLAP) in FIL-ASPCs versus CON-ASPCs. Furthermore, qPCR confirmed that the expression levels of these genes were significantly upregulated in FIL-ASPCs (Fig. 4G**–**L). Integrative genomics viewer (IGV) track visualization confirmed pathway-specific H3K18la accumulation at promoter regions of GO-annotated genes, revealing significantly higher H3K18la occupancy at promoter regions of these genes in FIL-ASPCs compared to CON-ASPCs (Figure S3A**–**D). Correlation analysis demonstrated that the expression of pro-osteogenic differentiation genes, such as ALPL, RUNX2, IBSP, and BMP4, was positively associated with intracellular lactate levels (Fig. 4M**–**P). Collectively, these observations suggested that H3K18 lactylation epigenetically potentiating ASPC osteogenic capacity through coordinated transcriptional activation of skeletal developmental programs.

### TGF-β1 promoted histone lactylation and osteogenesis in FIL-ASPCs

**Fig. 5:**
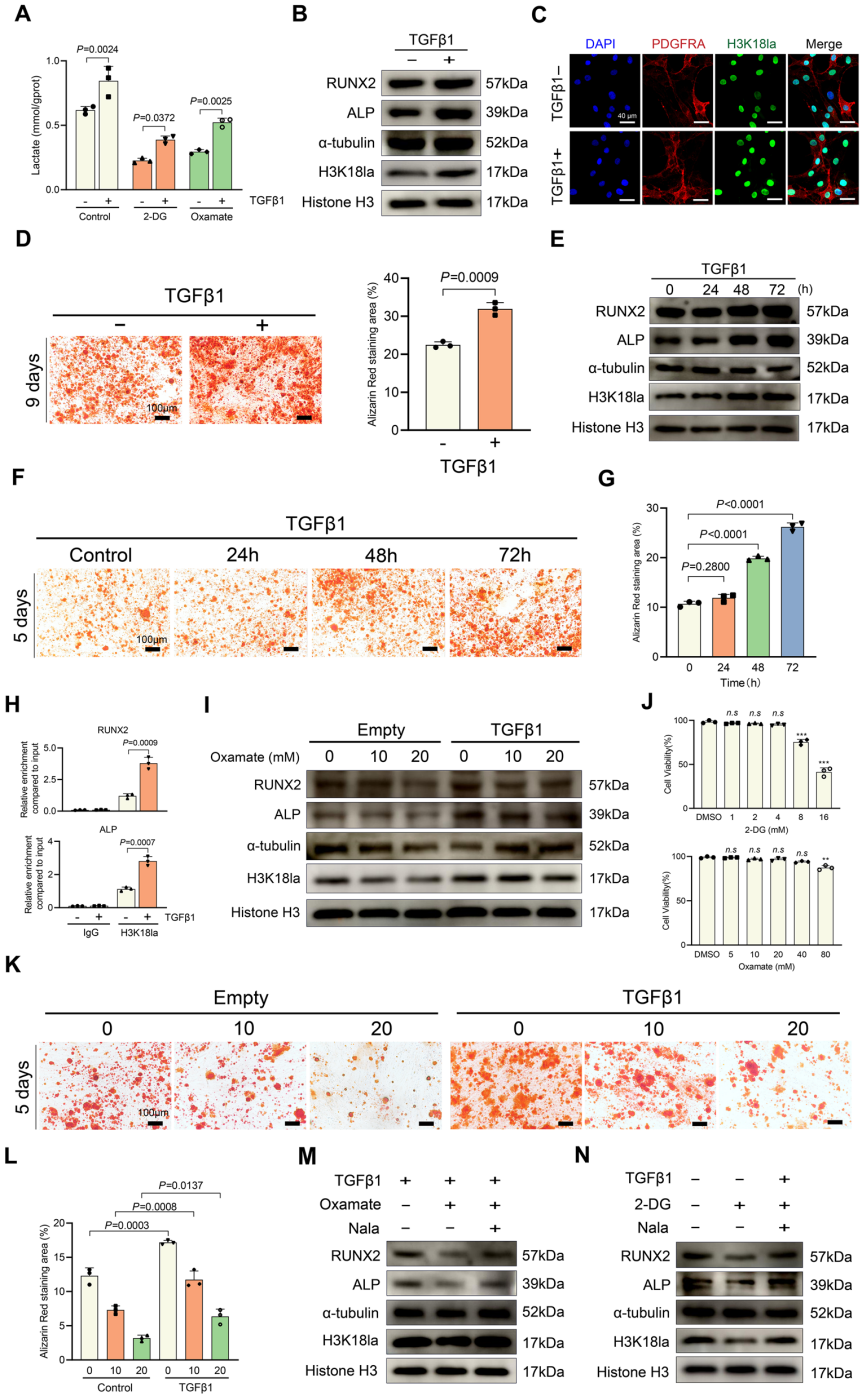
TGF-β1 promoted H3K18la level and osteogenesis of FIL-ASPCs. **A** Measurement of lactate production in FIL-ASPCs after TGF-β1 (1 ng/ml) and 4mM 2-DG and 20mM oxamate treatment for 24 h. **B** H3K18la, RUNX2, and ALP levels were analyzed by Western blotting in FIL-ASPCs treated with TGF-β1 or not. **C** H3K18la levels were visualized by immunofluorescence staining in FIL-ASPCs treated with TGF-β1 or not. **D** ARS of FIL-ASPCs treated with TGF-β1 or not. **E** RUNX2, ALP, and H3K18la levels in FIL-ASPCs treated with TGFβ1 (1 ng/ml) at the indicated time points were analyzed by Western blotting. **F**, **G**: ARS of FIL-ASPCs treated with TGFβ1 (1 ng/ml) at the indicated time points after osteogenic medium administration. **H** ChIP‒qPCR analysis of H3K18la at the RUNX2 and ALP promoter in FIL-ASPCs treated with TGFβ1 (1 ng/ml) for 24 h. Chromatin was immunoprecipitated using anti-H3K18la or anti-IgG antibodies, and quantification was performed by qPCR. **I** RUNX2, ALP, and H3K18la levels in FIL-ASPCs treated with TGF-β1 and Oxamate were evaluated by Western blotting. The FIL-ASPCs were treated with 10 or 20 mM Oxamate with TGFβ1 (1 ng/ml) for 3days. **J** Cell viability assays in FIL-ASPCs treated with 2-DG or oxamate at the indicated concentrations for 24 h. **K**, **L** ARS of FIL-ASPCs treated with TGFβ1 and oxamate after osteogenic medium administration. **M**, **N** RUNX2, ALP and H3K18la levels treated with TGF-β1, oxamate and Nala simultaneously were measured by Western blotting. Data were analyzed by unpaired two-sided Student’s t tests (**D**) or one-way ANOVA (**G**, **H**, **J**, **L**) and were presented as mean ± SD with three replicate experiments (biological replicates). ASPCs isolated from FIL patients No. 1–3 and CON patients No. 14–16 were used for Western blotting and osteogenic induction. Full-length blots were presented in Figure S5

The pleiotropic cytokine TGF-β1, a well-established regulator of mesenchymal stem cell osteogenic commitment [15], has recently been implicated in metabolic reprogramming through glycolytic pathway activation in stromal cells [16]. KEGG analysis revealed that the upregulated peak-related genes were enriched in TGFβ signaling pathway in FIL-ASPCs (Fig. 4F). To delineate TGF-β1’s regulatory role in this pathological context, we assessed its metabolic and epigenetic impacts. Pharmacological stimulation with TGF-β1 induced substantial lactate accumulation in FIL-ASPCs, an effect abrogated by concurrent administration of glycolytic suppressors (2-DG or oxamate) (Fig. 5A). Western blot and immunofluorescence analyses validated TGF-β1-induced elevation of H3K18 lactylation (Figs. 5B, C), which coincided with accelerated osteogenic commitment (Figs. 5B, D). Temporal profiling indicated a gradual accumulation of H3K18la with extended TGF-β1 exposure, paralleling the sequential upregulation of osteogenic transcriptional markers (Fig. 5E). ARS staining further demonstrated a time-dependent increase in osteogenic potential in FIL-ASPCs under continuous TGF-β1 stimulation (Fig. 5F, G). To uncover the epigenetic mechanisms driving TGF-β1-mediated gene activation, we conducted ChIP-qPCR to examine histone lactylation at the promoter regions. In line with our immunoblot findings, TGF-β1 significantly elevated H3K18la levels at the RUNX2 and ALP promoters (Fig. 5H).

Subsequently, we explored whether TGF-β1 could mitigate the impact of glycolytic inhibitors. Increasing the concentration of oxamate from 10 mM to 20 mM significantly diminished H3K18 lactylation levels. Nevertheless, TGF-β1 treatment effectively restored H3K18la modification even at both elevated and lower oxamate concentrations (Fig. 5I). In line with its osteogenic-promoting function, TGF-β1 upregulated the expression of RUNX2 and ALP while also enhancing matrix mineralization (Fig. 5I, K, L). Further experiments revealed that combined inhibition using 2-DG and oxamate not only suppressed H3K18la but also reversed the TGF-β1-induced upregulation of RUNX2 and ALP (Fig. 5M, N). Cell viability assays indicated that these effects were not due to cytotoxicity (Fig. 5J). Importantly, supplementation with exogenous lactate rescued the impaired activation of osteogenic markers caused by glycolytic inhibitors (Fig. 5M, N). Collectively, these results demonstrated that TGF-β1 enhanced osteogenic differentiation in ASPCs via the upregulation of H3K18 lactylation.

### Feedback loop driven by H3K18la and HK2 promoted osteogenesis

**Fig. 6:**
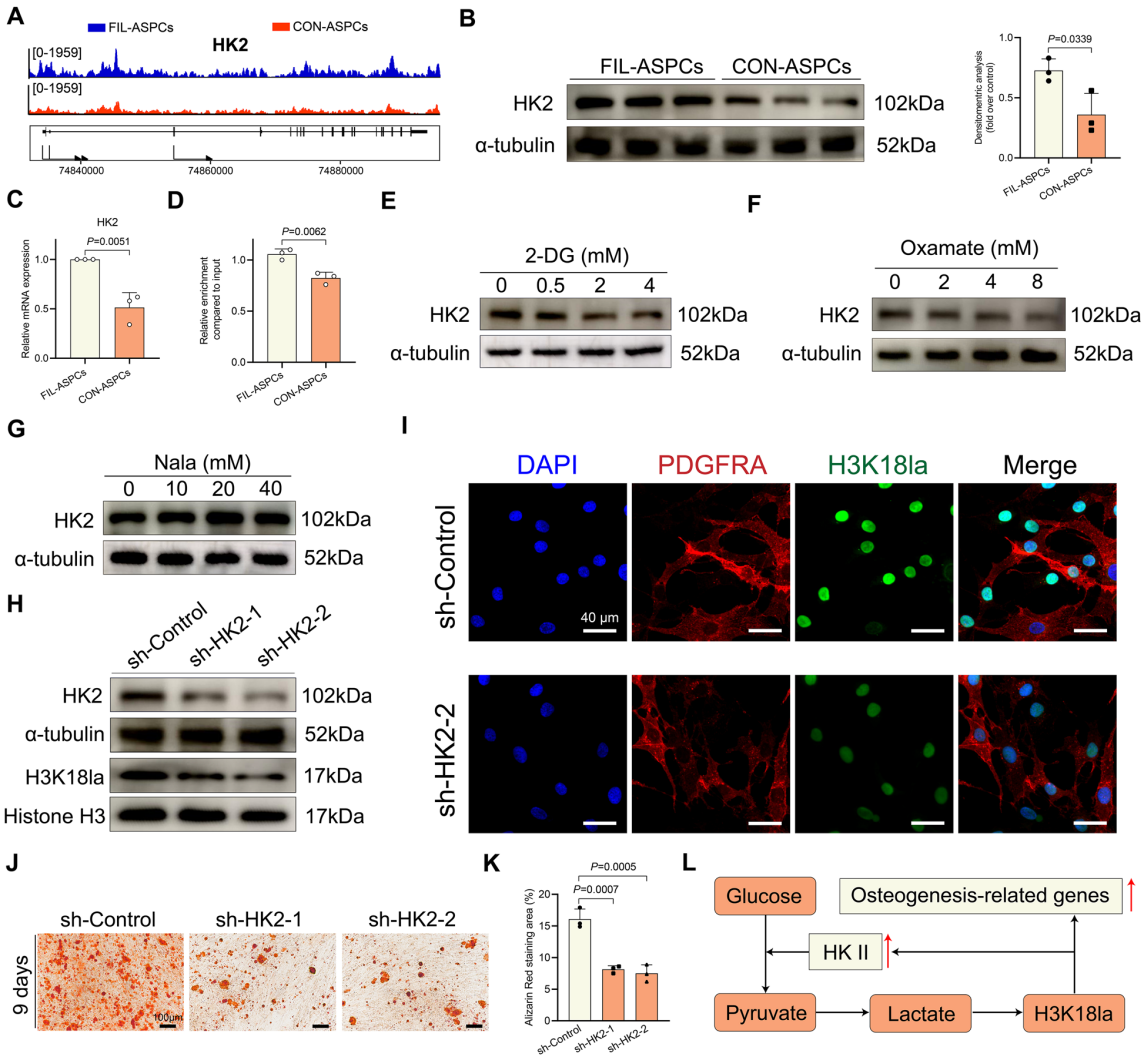
A feedback loop driven by H3K18la and HK2 promoted osteogenesis. **A** Genome browser tracks of CUT& Tag signal at the HK2 loci. **B** Western blot analysis of the expression of HK2 in FIL-ASPCs and CON- ASPCs. **C** The mRNA level of HK2 in FIL-ASPCs and CON- ASPCs. **D** ChIP‒qPCR analysis of H3K18la at the HK2 promoter in FIL-ASPCs and CON-ASPCs. **E**–**G** The expression of HK2 was determined by Western blot in 2-DG (**E**), oxamate (**F**), or Nala (**G**) treated FIL-ASPCs. H: The HK2 and H3K18la was determined by Western blot in FIL-ASPCs with or without HK2 knockdown. **I** H3K18la levels were visualized by immunofluorescence staining in FIL-ASPCs with or without HK2 knockdown. **J**, **K**: ARS of FIL-ASPCs with or without HK2 knockdown after osteogenic induction. **L** Schematic of the feedback loop driven by H3K18la and HK2. ASPCs isolated from FIL patients No. 1–3 and CON patients No. 14–16 were used for Western blotting and osteogenic induction. Data were analyzed by unpaired two-sided Student’s t tests (**B**, **C**, **D**) or one-way ANOVA (**K**) and were presented as mean ± SD with three replicate experiments (biological replicates). Full-length blots were presented in Figure S5

Interestingly, CUT& Tag analysis unveiled an increase in levels of H3K18la at the promotors of HK2 in FIL-ASPCs (Fig. 6A). We observed an increase in the expression of HK2 at both the mRNA and protein levels in FIL-ASPCs (Fig. 6B, C). ChIP-qPCR analysis also revealed that hyperlactylation of H3K18 increased its binding to the promoters of HK2 (Fig. 6D). HK2 is a crucial enzyme in glycolysis, responsible for converting glucose into glucose-6-phosphate. To investigated whether H3K18la promoted HK2 transcription, we treated FIL-ASPCs with different lactylation-modulating agents. Both 2-DG and oxamate reduced HK2 expression, while Nala slightly increased HK2 levels (Fig. 6E**–**G). These findings suggested that H3K18la was involved in regulating HK2 expression. To confirm the functional impact of the HK2/Lactate/H3K18la feedback loop on FIL-ASPCs, we knockdown HK2 in FIL-ASPCs using lentivirus (Fig. 6H). We observed that knockdown of HK2 resulted in significant decrease in H3K18 lactylation (Fig. 6H, I). Consistent with our previous results showing that H3K18la promoted osteogenesis, we found that HK2 knockdown inhibited FIL-ASPCs osteogenesis (Fig. 6J**–**K). Taken together, we confirmed that there existed a feedback loop in which lactate increased H3K18la and then promoted HK2 expression; increased HK2 expression in turn promoted H3K18la and elevated the expression of the downstream osteogenic genes (Fig. 6L).

## Discussion

**Fig. 7:**
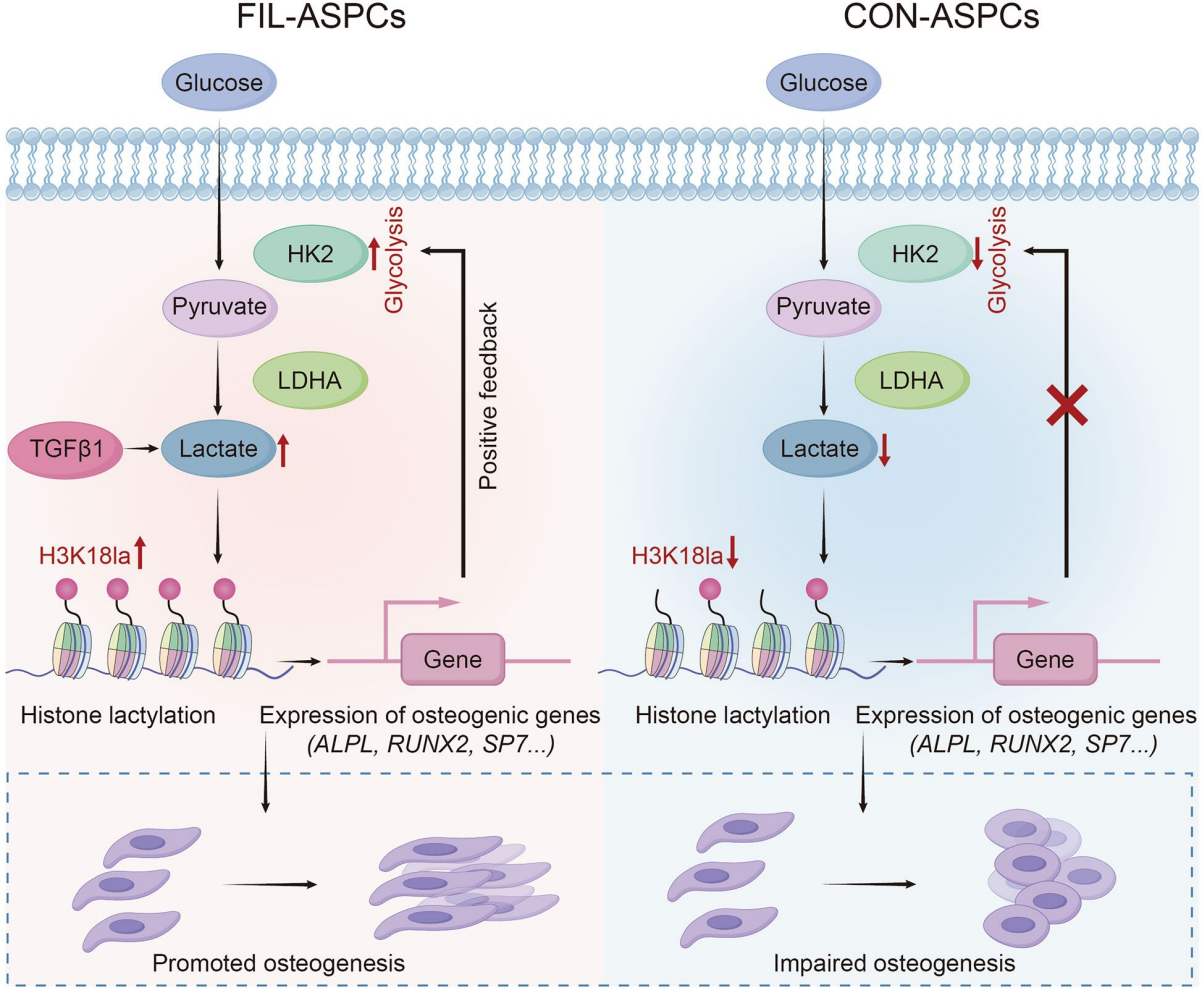
Schematic diagram of the molecular mechanism. In FIL-ASPCs, higher H3K18la level increased the expression of osteogenic genes and HK2. HK2 further promoted glycolysis, forming an H3K18la-lactate-H3K18la-HK2 feedback loop. Moreover, TGF-β1 can also increase the intracellular lactate and lactylation levels

Histone lactylation, an emerging epigenetic modification, has been shown to play a critical role in regulating gene expression and cellular metabolism [17]. Our findings revealed that elevated intracellular lactate levels in FIL-ASPCs, driven by PIK3CA mutation-associated metabolic reprogramming, induced hyperlactylation of H3K18. This epigenetic modification facilitated the transcriptional activation of osteogenic genes, including RUNX2, ALPL, and others, while simultaneously upregulating HK2 expression. Enhanced HK2 activity further amplified glycolysis and lactate production, establishing a self-reinforcing cycle that perpetuates osteogenesis (Fig. 7). These results align with emerging evidence linking histone lactylation to metabolic-epigenetic crosstalk in cellular differentiation [18]. For instance, recent studies demonstrated that lactate-derived histone lactylation regulated macrophage polarization and osteoblast osteogenesis, underscoring its broad regulatory potential in pathological contexts [19, 20]. Our work extended this paradigm to FIL, providing mechanistic insights into how metabolic dysregulation and epigenetic modifications synergistically drove osseous hyperplasia.

While our study demonstrated that H3K18la promoted osteogenesis of FIL-ASPCs, it is important to address the limitation that ASPCs were isolated from adipose tissue rather than directly from hyperplastic bone or BMSCs. This raises the question of whether the observed lactylation-driven osteogenesis in ASPCs fully recapitulates the pathological bone overgrowth in FIL. We acknowledge that BMSCs are the canonical osteoprogenitors in skeletal development, and their absence in our model may limit direct extrapolation to bony hyperplasia [21]. However, several lines of evidence support the relevance of ASPCs in FIL pathogenesis. First, FIL is characterized by concurrent adipose hyperplasia and osseous overgrowth, with somatic PIK3CA mutations detected in both tissues, suggesting a shared progenitor pool or crosstalk between adipogenic and osteogenic lineages [22]. Second, ASPCs exhibit remarkable plasticity under pathological conditions, and metabolic reprogramming induced by PIK3CA mutations may license ASPCs to adopt osteogenic fates [23]. Notably, our CUT&Tag and functional assays revealed that H3K18la directly activated osteogenic genes in ASPCs, mirroring transcriptional programs typically driven by BMSCs. While bone formation was not observed in adipose tissue under physiological conditions, the aberrant metabolic-epigenetic axis in FIL-ASPCs—fueled by lactate overproduction—likely overrides niche-specific constraints, enabling ectopic osteogenesis [24]. Future studies comparing ASPCs and BMSCs from FIL patients, or employing co-culture systems to model adipose-bone crosstalk, will further clarify the relative contributions of these cell types to skeletal pathology. Nonetheless, our findings highlight ASPCs as unexpected but plausible mediators of FIL-associated osseous hyperplasia, expanding the conceptual framework of mesenchymal cell plasticity in overgrowth disorders.

TGF-β1 is a well-known regulator of osteogenic differentiation and has been implicated in various metabolic processes, including glycolysis [25–27]. Our study demonstrated that TGF-β1 significantly enhanced histone lactylation and promoted osteogenesis in FIL-ASPCs. This effect was mediated through the upregulation of H3K18la, which in turn activated the expression of osteogenic markers. The ability of TGF-β1 to counteract the inhibitory effects of glycolytic inhibitors further underscored the importance of lactate metabolism in this process. These findings suggest that TGF-β1 may serve as a critical link between metabolic reprogramming and osteogenic differentiation in FIL.

The identification of a positive feedback loop involving H3K18la and HK2 represents a significant discovery in this study. We showed that H3K18la enhanced HK2 expression, which in turn promoted glycolysis and lactate accumulation, further increasing H3K18la levels. This feedback loop appears to be crucial for the sustained activation of osteogenic genes in FIL-ASPCs. Similar feedback mechanisms have been observed in other contexts. For example, the glycolysis/H4K12la/PKM2 positive feedback loop exacerbates microglial cell dysfunction, while H3K9la inhibits the expression of the lactylation eraser HDAC2, further aggravating H3K9la-induced angiogenic gene expression [28, 29]. Our findings suggest that targeting this feedback loop, either by inhibiting HK2 or modulating histone lactylation, could provide a novel therapeutic strategy to mitigate the abnormal osteogenesis in FIL.

Despite these advances, several limitations warrant consideration. First, the study’s reliance on a small cohort of FIL necessitates validation in larger, diverse populations to confirm the universality of the H3K18la-HK2 axis. Second, while in vitro experiments robustly demonstrate the role of lactylation in osteogenesis, in vivo evidence remains preliminary. We cannot clearly determine whether the effect of lactylation on bone healing in vivo is due to the osteogenic differentiation of the cells themselves or the cytokines they secrete. Future studies employing conditional knockout models or pharmacological inhibitors of lactylation in FIL animal models could strengthen translational relevance. Additionally, the interplay between H3K18la and other histone modifications (e.g., acetylation, methylation) in regulating osteogenic programs remains unexplored [30]. Given the complexity of epigenetic regulation, multi-omics approaches may uncover synergistic or antagonistic interactions that refine our understanding of FIL pathogenesis.

In conclusion, our study elucidates the critical role of histone lactylation, particularly H3K18la, in the pathogenesis of FIL. The findings highlight the complex interplay between metabolic reprogramming and epigenetic modifications in driving disease progression. Future research should focus on further characterizing the molecular mechanisms underlying this process and exploring the potential therapeutic applications of targeting histone lactylation and the H3K18la-HK2 feedback loop. Additionally, the identification of upstream regulators and downstream effectors of H3K18la in FIL-ASPCs may provide additional targets for intervention. Given the limited treatment options currently available for FIL, our study offers new avenues for developing targeted therapies to improve the quality of life for affected patients (Figure S4).

## Supplementary Information

Supplementary material 1. Figure S1: Characterization of primary ASPCs. A: The expression of the ASPCs surface markers CD73, CD90, and PDGFRA, the haematopoietic marker CD45 and the immune marker HLA-DR in isolated ASPCs at passage 1 was detected by flow cytometry. Figure S2: Oxamate and Nala had no effect on adipogenesis. A: The mature adipocytes with lipid droplets were visualized by Oil Red O staining on day 8 after 2-DG treatment. B: The expression of adipogenic marker genes PPAR γ, C/EBP α, and FABP4 was determined by Western blot in 2-DG treated FIL-ASPCs on day 3 of adipogenic differentiation. C: The mature adipocytes with lipid droplets were visualized by Oil Red O staining on day 8 after oxamate treatment. D: The expression of adipogenic marker genes PPAR γ, C/EBP α, and FABP4 was determined by Western blot in oxamate treated FIL-ASPCs on day 3 of adipogenic differentiation. E: The mature adipocytes with lipid droplets were visualized by Oil Red O staining on day 8 after Nala treatment. F: The expression of adipogenic marker genes PPAR γ, C/EBP α, and FABP4 was determined by Western blot in Nala treated FIL-ASPCs on day 3 of adipogenic differentiation. Full-length blots were presented in Figure S5. Figure S3: H3K18la activated the transcription of multiple genes related to osteogenesis. A: Genome browser tracks of CUT& Tag signal at the LEF1 and COL6A1 loci. B: Genome browser tracks of CUT& Tag signal at the ZBTB16 and FOXC1 loci. C: Genome browser tracks of CUT& Tag signal at the FGF18 and FBN1 loci. D: Genome browser tracks of CUT& Tag signal at the HAS2 and COL27A1 loci. Figure S4: Schematic diagram of the therapeutic strategies. Our research demonstrated that inhibiting HK2, LDH, and TGFβ1 may potentially suppress the abnormal osteogenic differentiation in FIL. Figure S5: Uncropped blot.

Supplementary material 2.

Supplementary material 3.

## Data Availability

The datasets used and/or analyzed during the current study are available in NCBI’s Gene Expression Omnibus (GEO) and are accessible through GEO Series accession number GSE296002 (CUT&Tag).
