# Supplementary material for: H3K18 lactylation-hexokinase 2 positive feedback loop promotes osteogenesis of ASPCs in facial infiltrating lipomatosis
Source: Stem Cell Res Ther. 2025 Oct 1;16:538. doi: 10.1186/s13287-025-04651-5 (PMC12486737; doi:10.1186/s13287-025-04651-5)
Supplement: Supplementary file 1 — Supplementary material 1. Figure S1: Characterization of primary ASPCs. A: The expression of the ASPCs surface markers CD73, CD90, and PDGFRA, the haematopoietic marker CD45 and the immune marker HLA-DR in isolated ASPCs at passage 1 was detected by flow cytometry. Figure S2: Oxamate and Nala had no effect on adipogenesis. A: The mature adipocytes with lipid droplets were visualized by Oil Red O staining on day 8 after 2-DG treatment. B: The expression of adipogenic marker genes PPAR γ, C/EBP α, and FABP4 was determined by Western blot in 2-DG treated FIL-ASPCs on day 3 of adipogenic differentiation. C: The mature adipocytes with lipid droplets were visualized by Oil Red O staining on day 8 after oxamate treatment. D: The expression of adipogenic marker genes PPAR γ, C/EBP α, and FABP4 was determined by Western blot in oxamate treated FIL-ASPCs on day 3 of adipogenic differentiation. E: The mature adipocytes with lipid droplets were visualized by Oil Red O staining on day 8 after Nala treatment. F: The expression of adipogenic marker genes PPAR γ, C/EBP α, and FABP4 was determined by Western blot in Nala treated FIL-ASPCs on day 3 of adipogenic differentiation. Full-length blots were presented in Figure S5. Figure S3: H3K18la activated the transcription of multiple genes related to osteogenesis. A: Genome browser tracks of CUT& Tag signal at the LEF1 and COL6A1 loci. B: Genome browser tracks of CUT& Tag signal at the ZBTB16 and FOXC1 loci. C: Genome browser tracks of CUT& Tag signal at the FGF18 and FBN1 loci. D: Genome browser tracks of CUT& Tag signal at the HAS2 and COL27A1 loci. Figure S4: Schematic diagram of the therapeutic strategies. Our research demonstrated that inhibiting HK2, LDH, and TGFβ1 may potentially suppress the abnormal osteogenic differentiation in FIL. Figure S5: Uncropped blot. [file 13287_2025_4651_MOESM1_ESM.zip › New folder/Figure S5-uncropped blot.pdf]

Uncropped western blotting analysis

Figure 1G

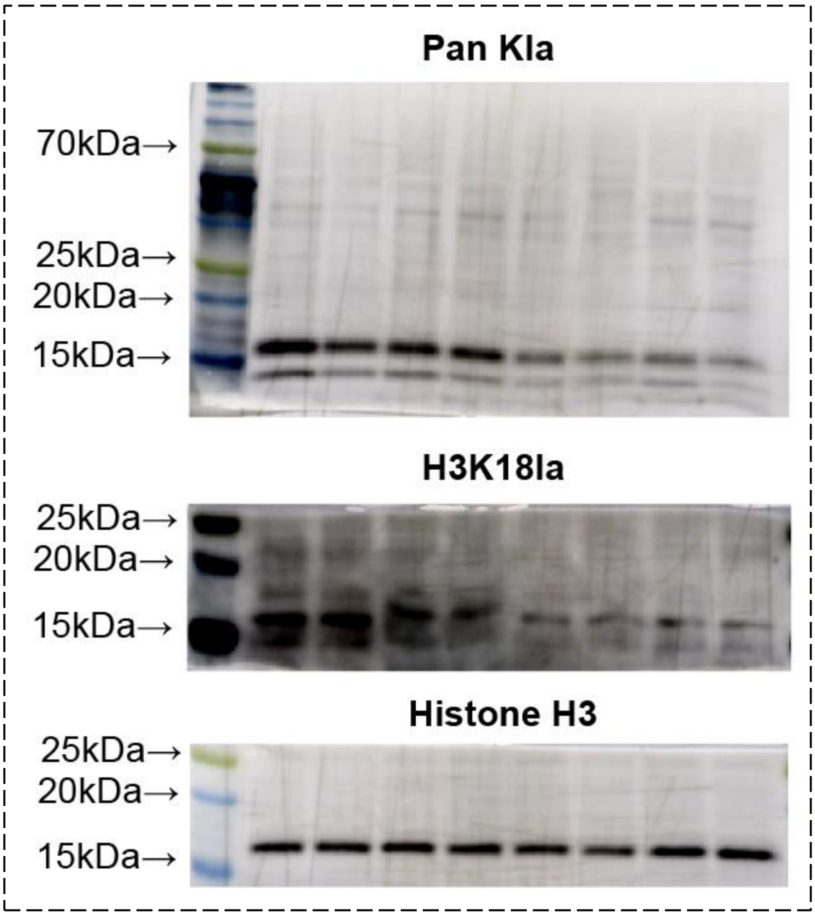

Figure 1I

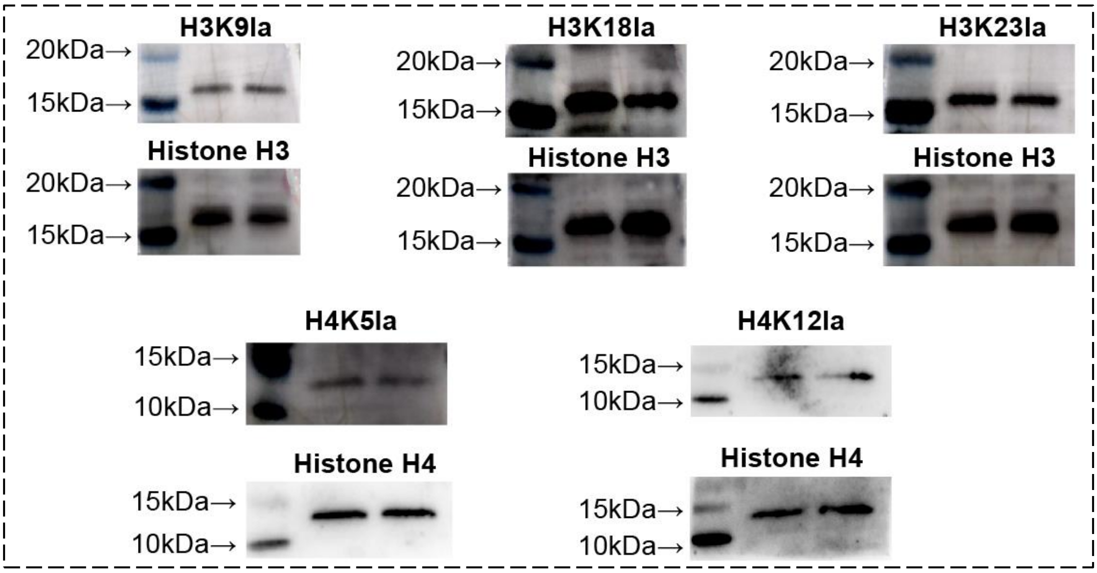

Figure 2E

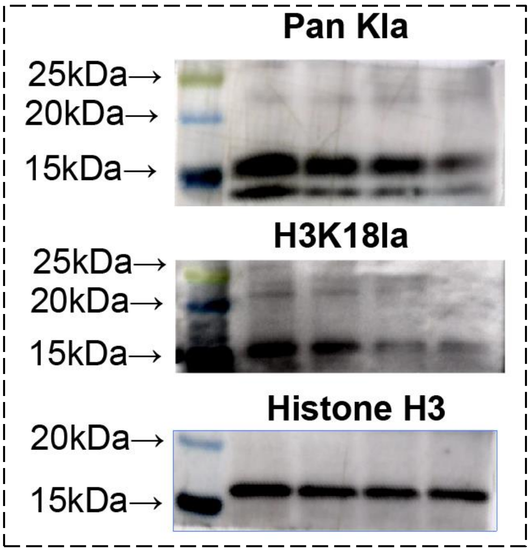

Figure 2F

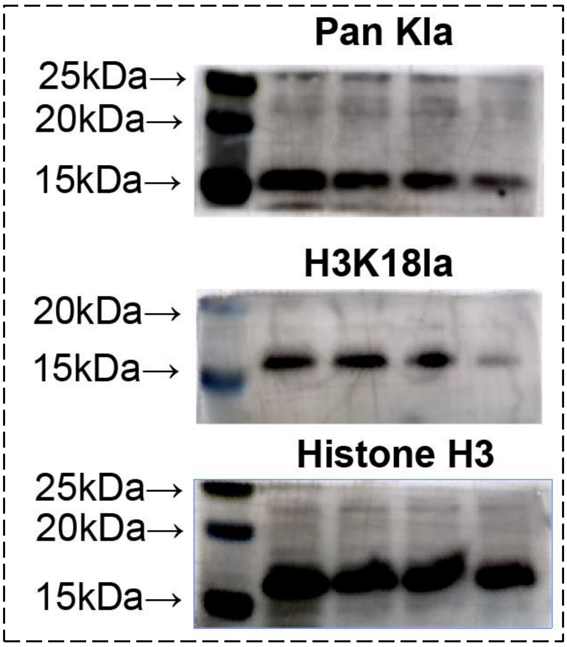

Figure 2G

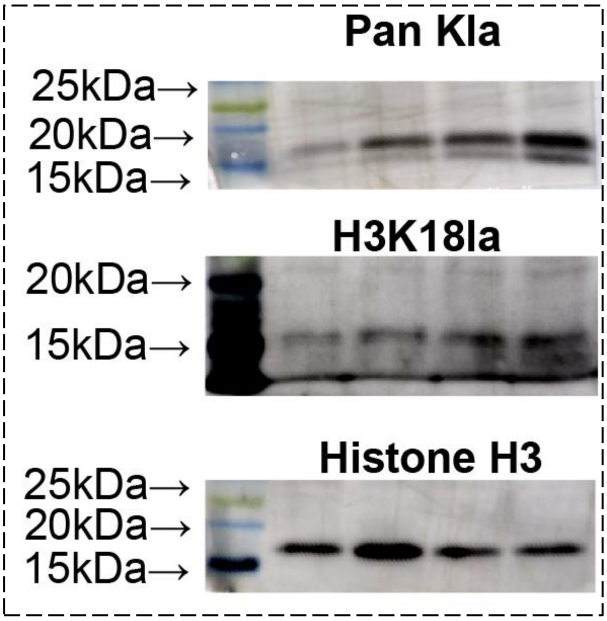

Figure 2H

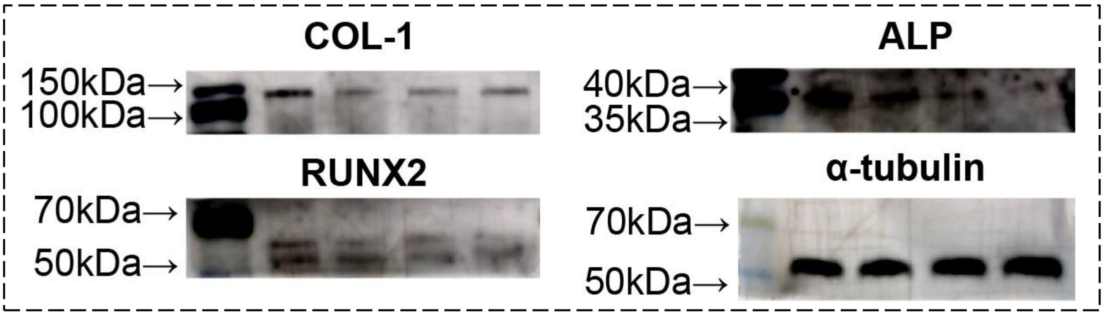

Figure 2I

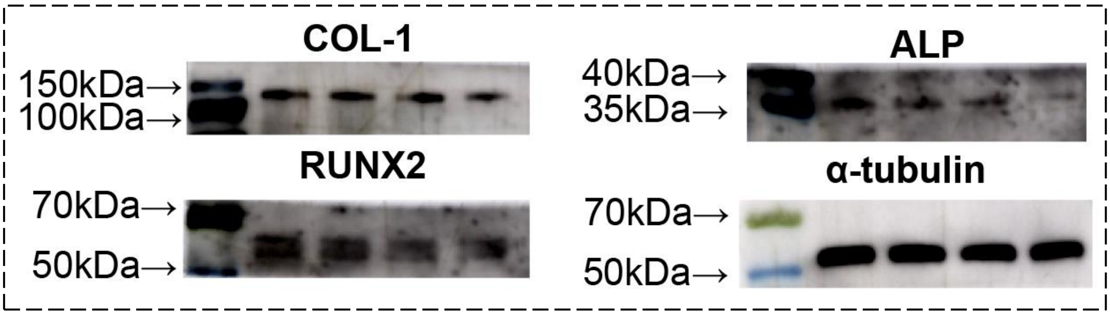

Figure 2J

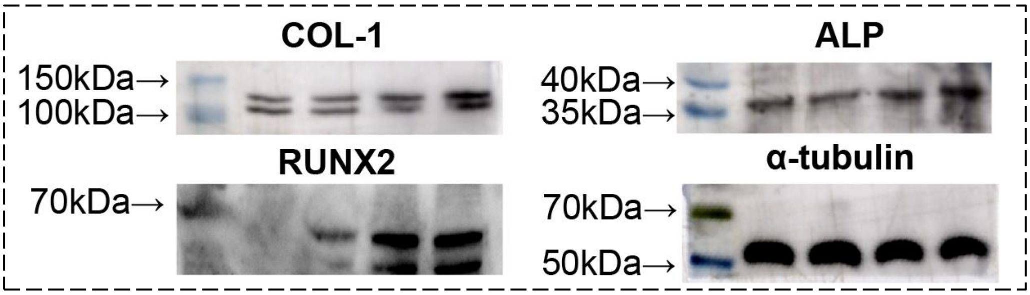

Figure 2N

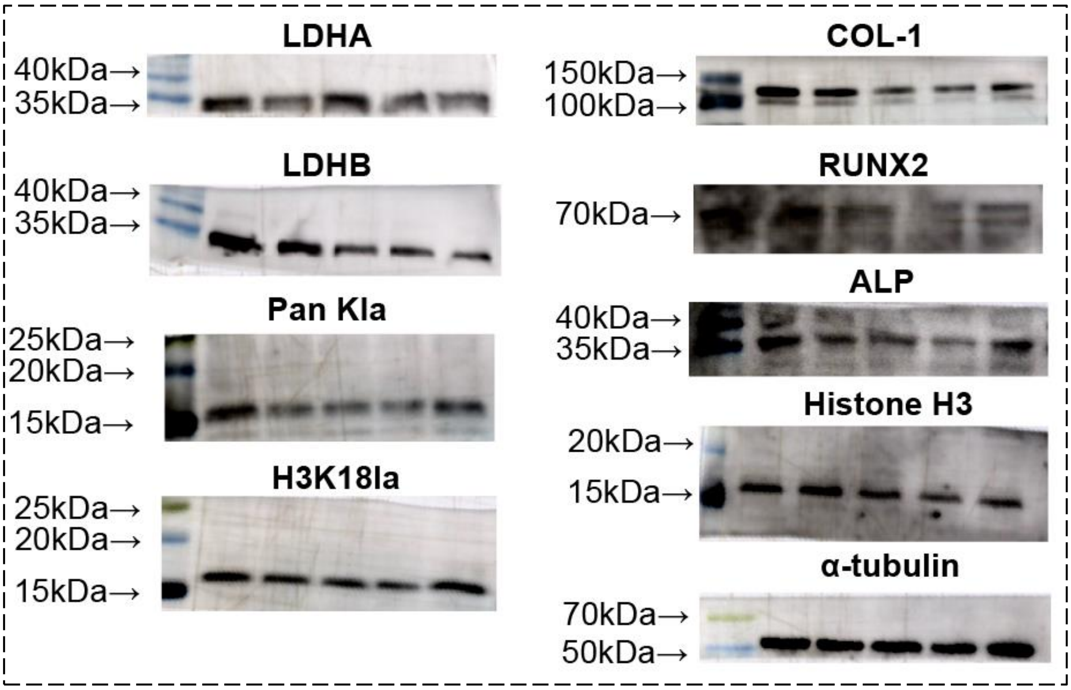

Figure 5B

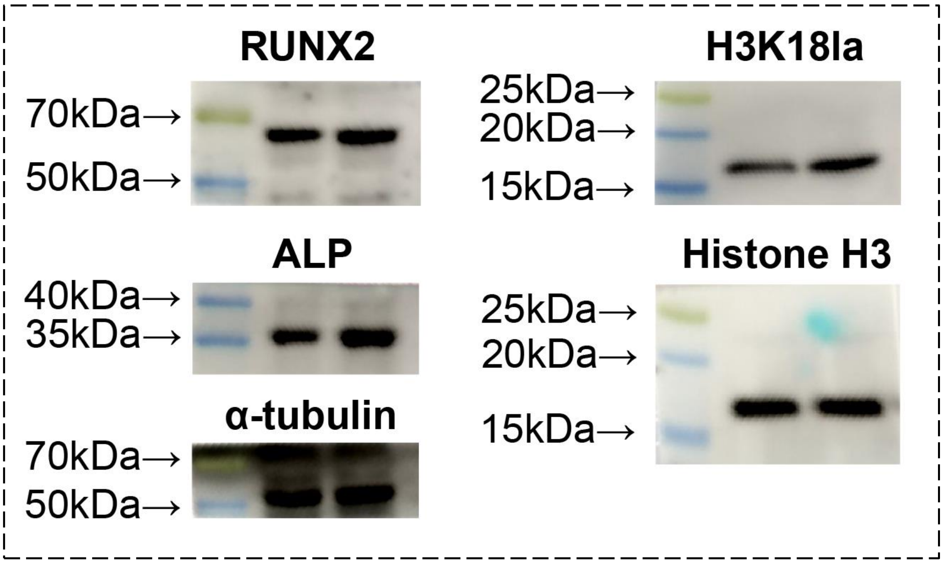

Figure 5E

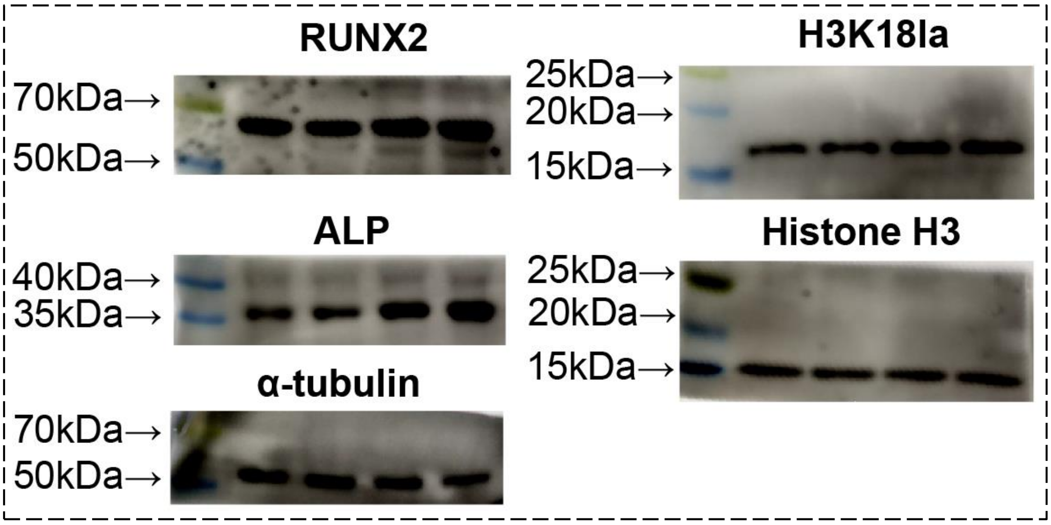

Figure 5I

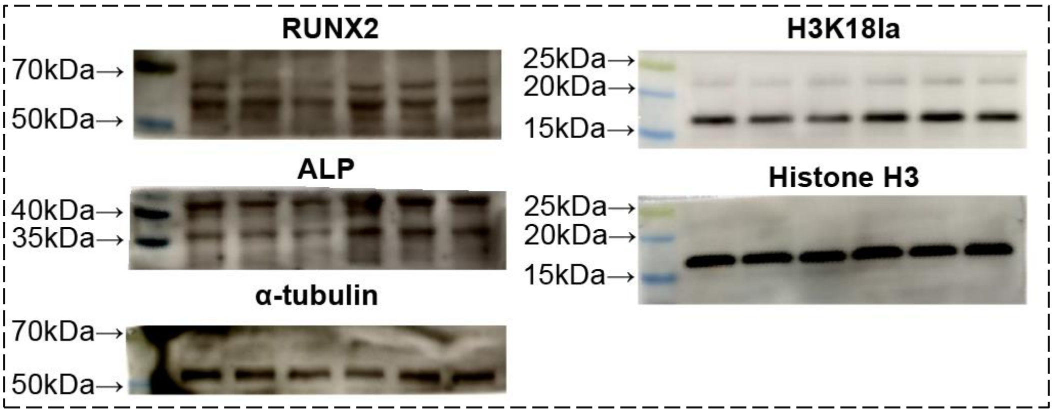

Figure 5M

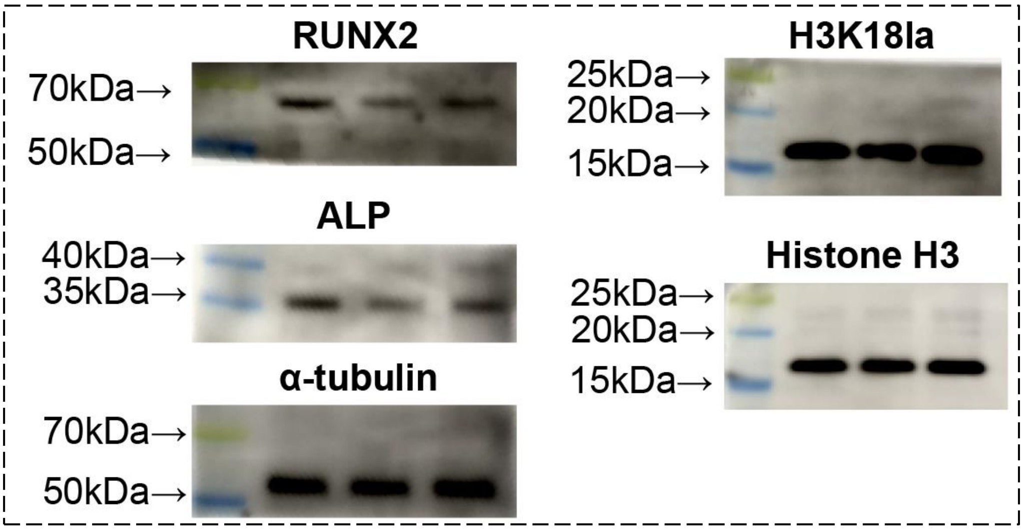

Figure 5N

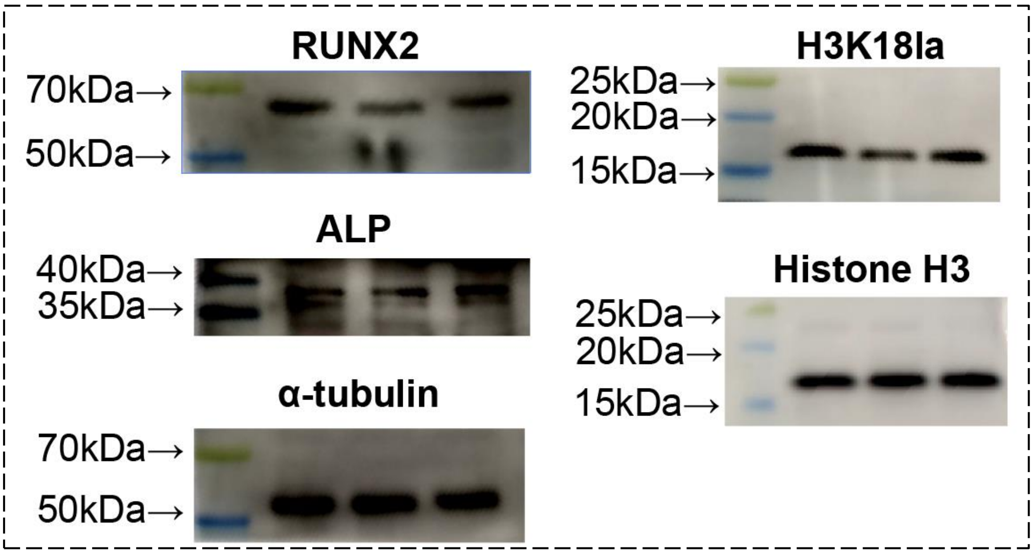

Figure 6B

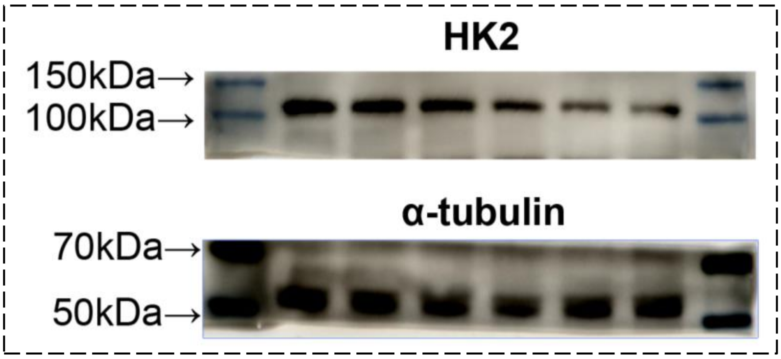

Figure 6E

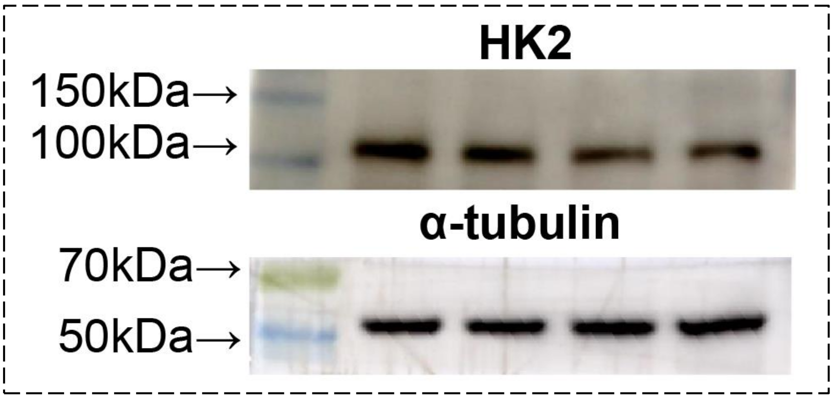

Figure 6F

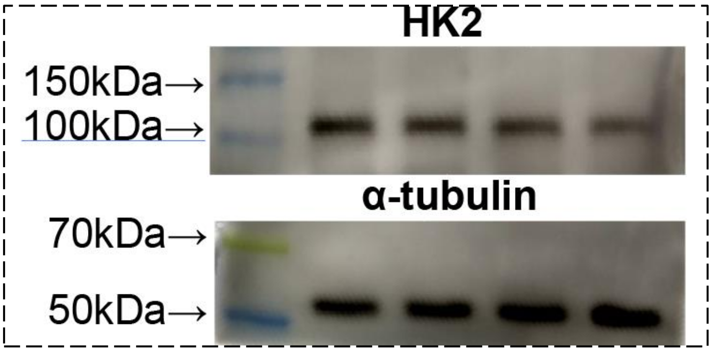

Figure 6G

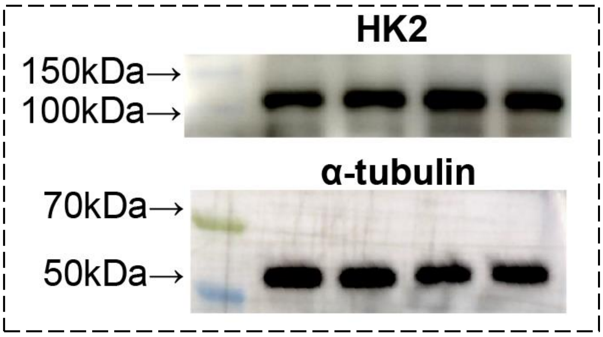

Figure 6H

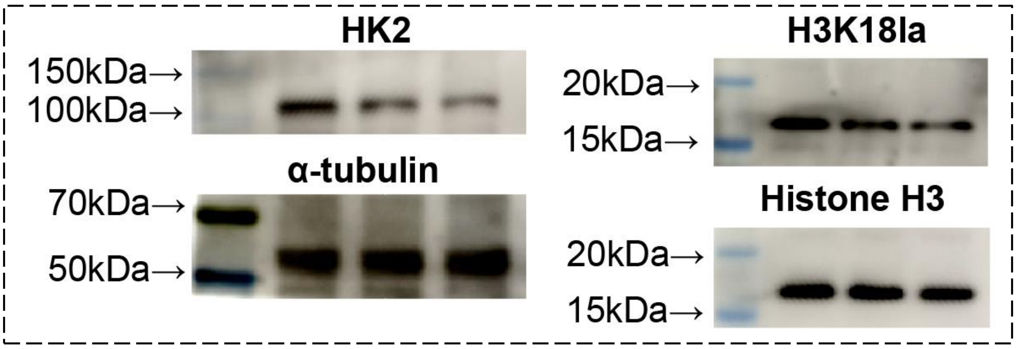

Figure S2B

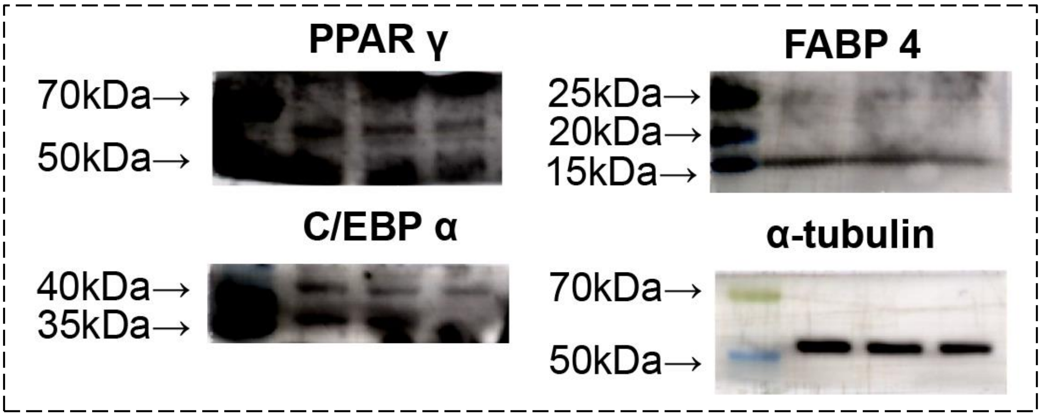

Figure S2D

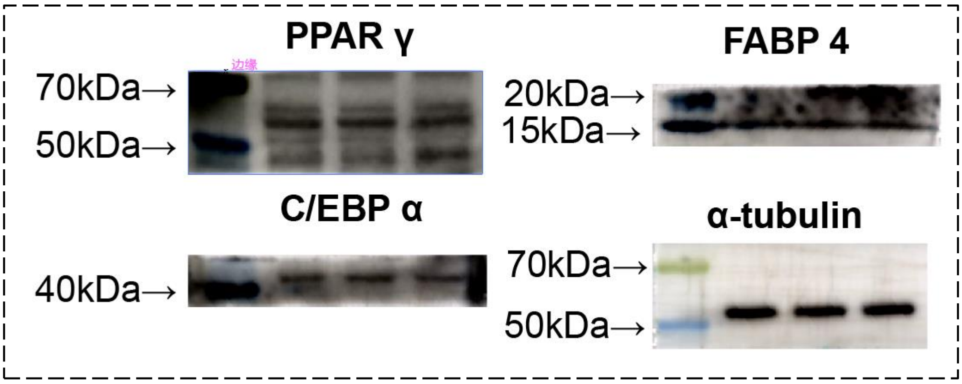
PPAR γ
70kDa→
50kDa→
FABP 4
20kDa→
15kDa→
C/EBP α
40kDa→
α-tubulin
70kDa→
50kDa→

Figure S2F

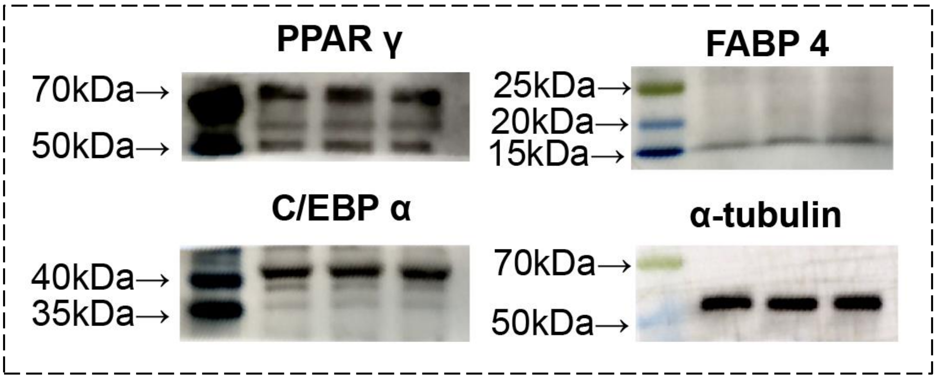
PPAR γ
70kDa→
50kDa→
FABP 4
25kDa→
20kDa→
15kDa→
C/EBP α
40kDa→
35kDa→
α-tubulin
70kDa→
50kDa→
