# Supplementary figures and images for: H3K18 lactylation-hexokinase 2 positive feedback loop promotes osteogenesis of ASPCs in facial infiltrating lipomatosis
Source: Stem Cell Res Ther. 2025 Oct 1;16:538. doi: 10.1186/s13287-025-04651-5 (PMC12486737; doi:10.1186/s13287-025-04651-5)

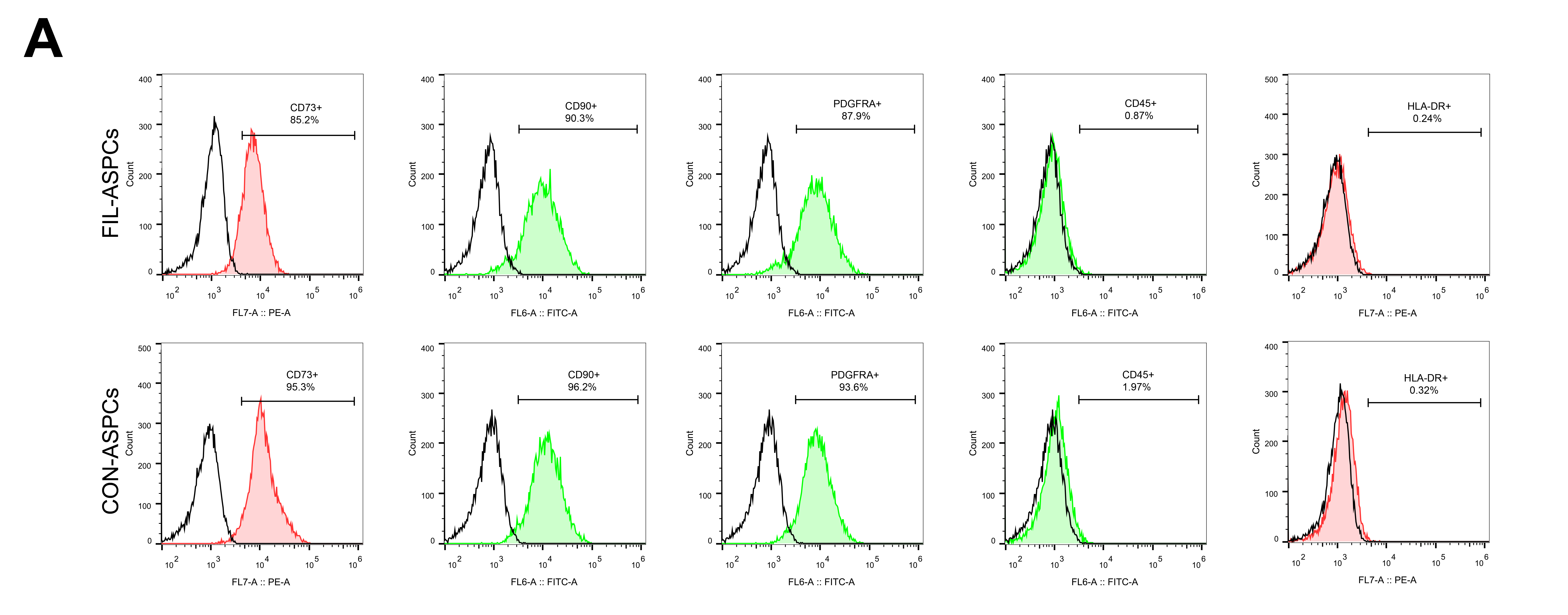

Supplement: Supplementary file 1 — Supplementary material 1. Figure S1: Characterization of primary ASPCs. A: The expression of the ASPCs surface markers CD73, CD90, and PDGFRA, the haematopoietic marker CD45 and the immune marker HLA-DR in isolated ASPCs at passage 1 was detected by flow cytometry. Figure S2: Oxamate and Nala had no effect on adipogenesis. A: The mature adipocytes with lipid droplets were visualized by Oil Red O staining on day 8 after 2-DG treatment. B: The expression of adipogenic marker genes PPAR γ, C/EBP α, and FABP4 was determined by Western blot in 2-DG treated FIL-ASPCs on day 3 of adipogenic differentiation. C: The mature adipocytes with lipid droplets were visualized by Oil Red O staining on day 8 after oxamate treatment. D: The expression of adipogenic marker genes PPAR γ, C/EBP α, and FABP4 was determined by Western blot in oxamate treated FIL-ASPCs on day 3 of adipogenic differentiation. E: The mature adipocytes with lipid droplets were visualized by Oil Red O staining on day 8 after Nala treatment. F: The expression of adipogenic marker genes PPAR γ, C/EBP α, and FABP4 was determined by Western blot in Nala treated FIL-ASPCs on day 3 of adipogenic differentiation. Full-length blots were presented in Figure S5. Figure S3: H3K18la activated the transcription of multiple genes related to osteogenesis. A: Genome browser tracks of CUT& Tag signal at the LEF1 and COL6A1 loci. B: Genome browser tracks of CUT& Tag signal at the ZBTB16 and FOXC1 loci. C: Genome browser tracks of CUT& Tag signal at the FGF18 and FBN1 loci. D: Genome browser tracks of CUT& Tag signal at the HAS2 and COL27A1 loci. Figure S4: Schematic diagram of the therapeutic strategies. Our research demonstrated that inhibiting HK2, LDH, and TGFβ1 may potentially suppress the abnormal osteogenic differentiation in FIL. Figure S5: Uncropped blot. [file 13287_2025_4651_MOESM1_ESM.zip › New folder/Figure S1.jpg]

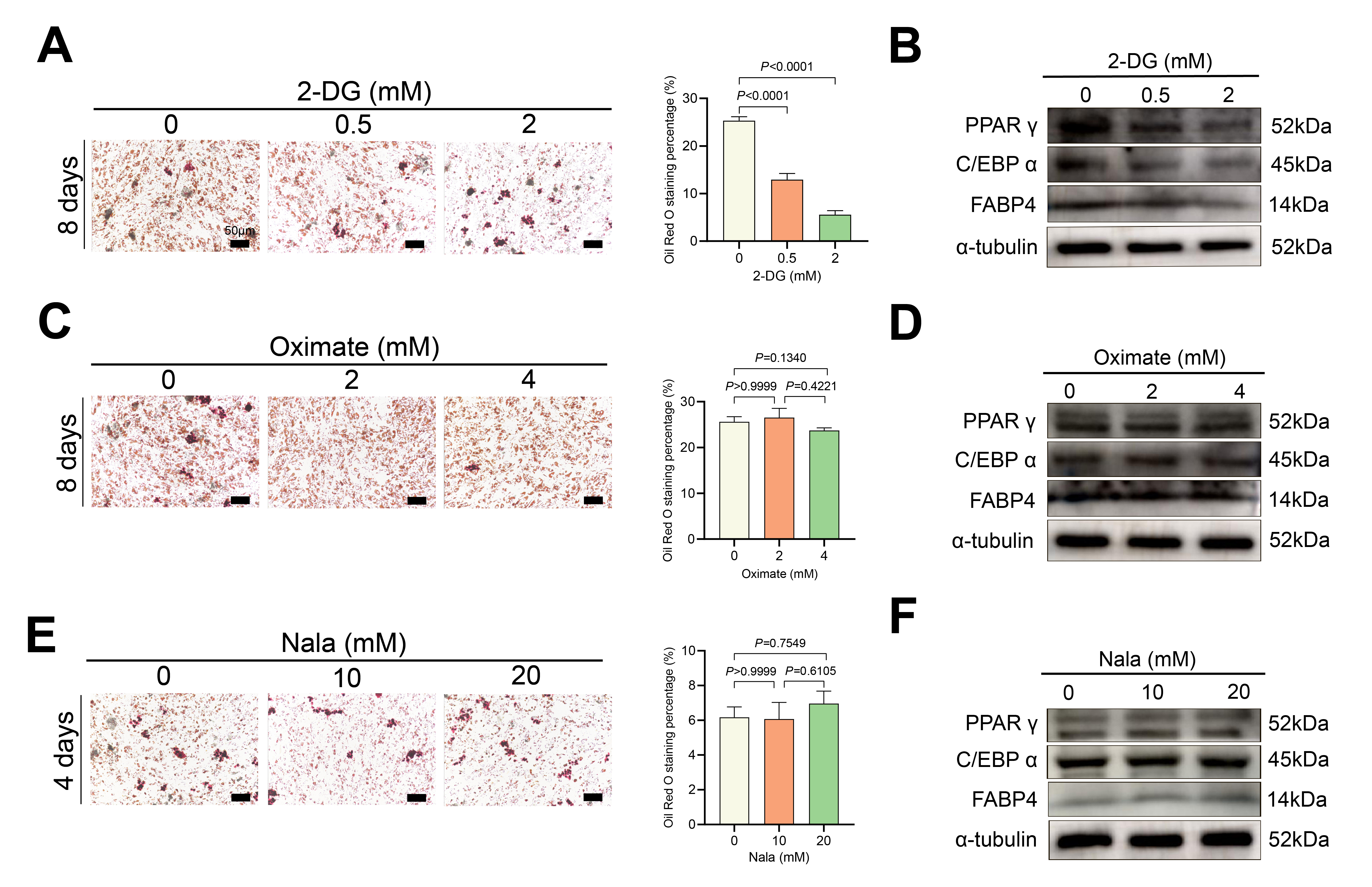

Supplement: Supplementary file 1 — Supplementary material 1. Figure S1: Characterization of primary ASPCs. A: The expression of the ASPCs surface markers CD73, CD90, and PDGFRA, the haematopoietic marker CD45 and the immune marker HLA-DR in isolated ASPCs at passage 1 was detected by flow cytometry. Figure S2: Oxamate and Nala had no effect on adipogenesis. A: The mature adipocytes with lipid droplets were visualized by Oil Red O staining on day 8 after 2-DG treatment. B: The expression of adipogenic marker genes PPAR γ, C/EBP α, and FABP4 was determined by Western blot in 2-DG treated FIL-ASPCs on day 3 of adipogenic differentiation. C: The mature adipocytes with lipid droplets were visualized by Oil Red O staining on day 8 after oxamate treatment. D: The expression of adipogenic marker genes PPAR γ, C/EBP α, and FABP4 was determined by Western blot in oxamate treated FIL-ASPCs on day 3 of adipogenic differentiation. E: The mature adipocytes with lipid droplets were visualized by Oil Red O staining on day 8 after Nala treatment. F: The expression of adipogenic marker genes PPAR γ, C/EBP α, and FABP4 was determined by Western blot in Nala treated FIL-ASPCs on day 3 of adipogenic differentiation. Full-length blots were presented in Figure S5. Figure S3: H3K18la activated the transcription of multiple genes related to osteogenesis. A: Genome browser tracks of CUT& Tag signal at the LEF1 and COL6A1 loci. B: Genome browser tracks of CUT& Tag signal at the ZBTB16 and FOXC1 loci. C: Genome browser tracks of CUT& Tag signal at the FGF18 and FBN1 loci. D: Genome browser tracks of CUT& Tag signal at the HAS2 and COL27A1 loci. Figure S4: Schematic diagram of the therapeutic strategies. Our research demonstrated that inhibiting HK2, LDH, and TGFβ1 may potentially suppress the abnormal osteogenic differentiation in FIL. Figure S5: Uncropped blot. [file 13287_2025_4651_MOESM1_ESM.zip › New folder/Figure S2.jpg]

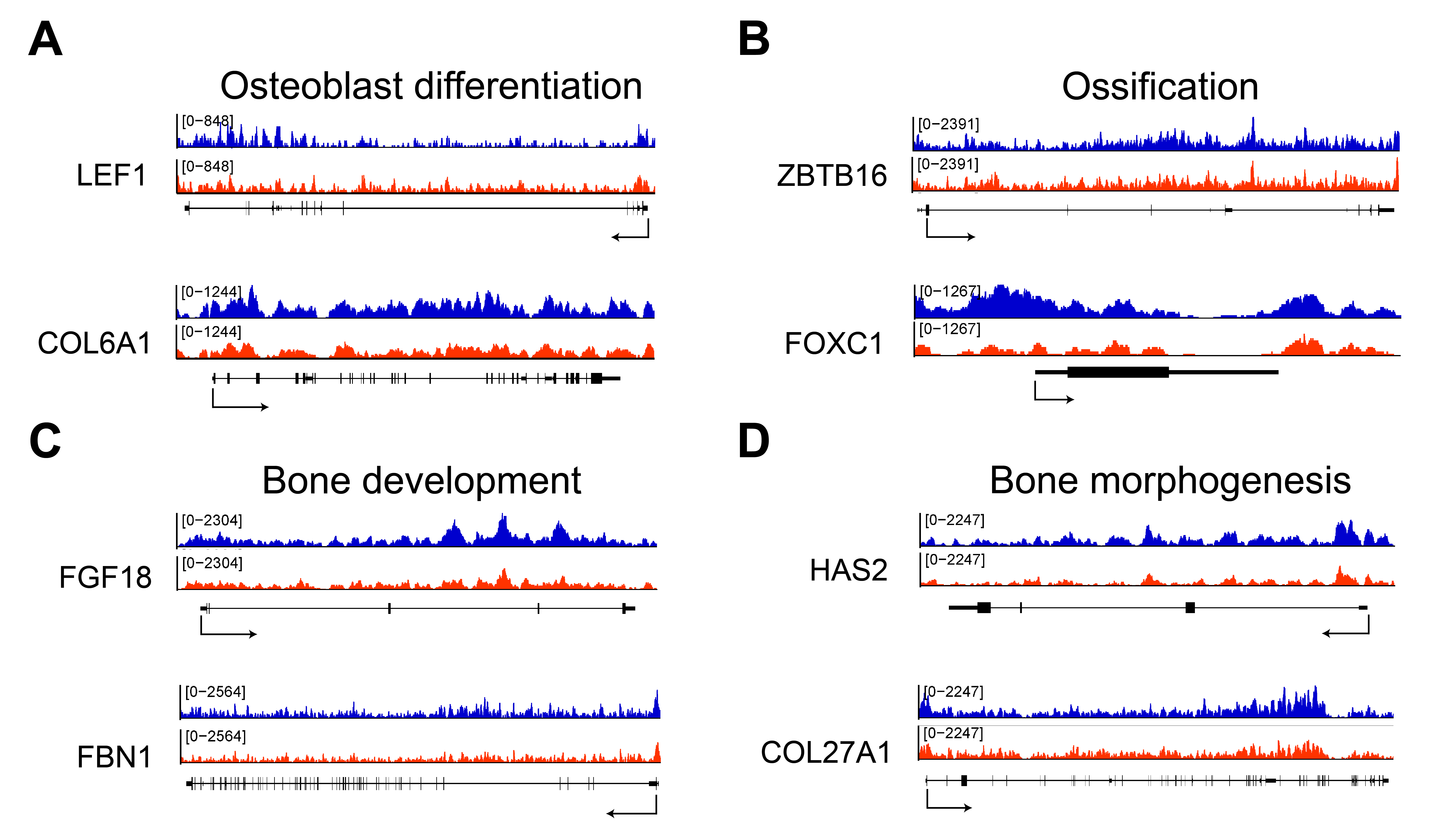

Supplement: Supplementary file 1 — Supplementary material 1. Figure S1: Characterization of primary ASPCs. A: The expression of the ASPCs surface markers CD73, CD90, and PDGFRA, the haematopoietic marker CD45 and the immune marker HLA-DR in isolated ASPCs at passage 1 was detected by flow cytometry. Figure S2: Oxamate and Nala had no effect on adipogenesis. A: The mature adipocytes with lipid droplets were visualized by Oil Red O staining on day 8 after 2-DG treatment. B: The expression of adipogenic marker genes PPAR γ, C/EBP α, and FABP4 was determined by Western blot in 2-DG treated FIL-ASPCs on day 3 of adipogenic differentiation. C: The mature adipocytes with lipid droplets were visualized by Oil Red O staining on day 8 after oxamate treatment. D: The expression of adipogenic marker genes PPAR γ, C/EBP α, and FABP4 was determined by Western blot in oxamate treated FIL-ASPCs on day 3 of adipogenic differentiation. E: The mature adipocytes with lipid droplets were visualized by Oil Red O staining on day 8 after Nala treatment. F: The expression of adipogenic marker genes PPAR γ, C/EBP α, and FABP4 was determined by Western blot in Nala treated FIL-ASPCs on day 3 of adipogenic differentiation. Full-length blots were presented in Figure S5. Figure S3: H3K18la activated the transcription of multiple genes related to osteogenesis. A: Genome browser tracks of CUT& Tag signal at the LEF1 and COL6A1 loci. B: Genome browser tracks of CUT& Tag signal at the ZBTB16 and FOXC1 loci. C: Genome browser tracks of CUT& Tag signal at the FGF18 and FBN1 loci. D: Genome browser tracks of CUT& Tag signal at the HAS2 and COL27A1 loci. Figure S4: Schematic diagram of the therapeutic strategies. Our research demonstrated that inhibiting HK2, LDH, and TGFβ1 may potentially suppress the abnormal osteogenic differentiation in FIL. Figure S5: Uncropped blot. [file 13287_2025_4651_MOESM1_ESM.zip › New folder/Figure S3.jpg]

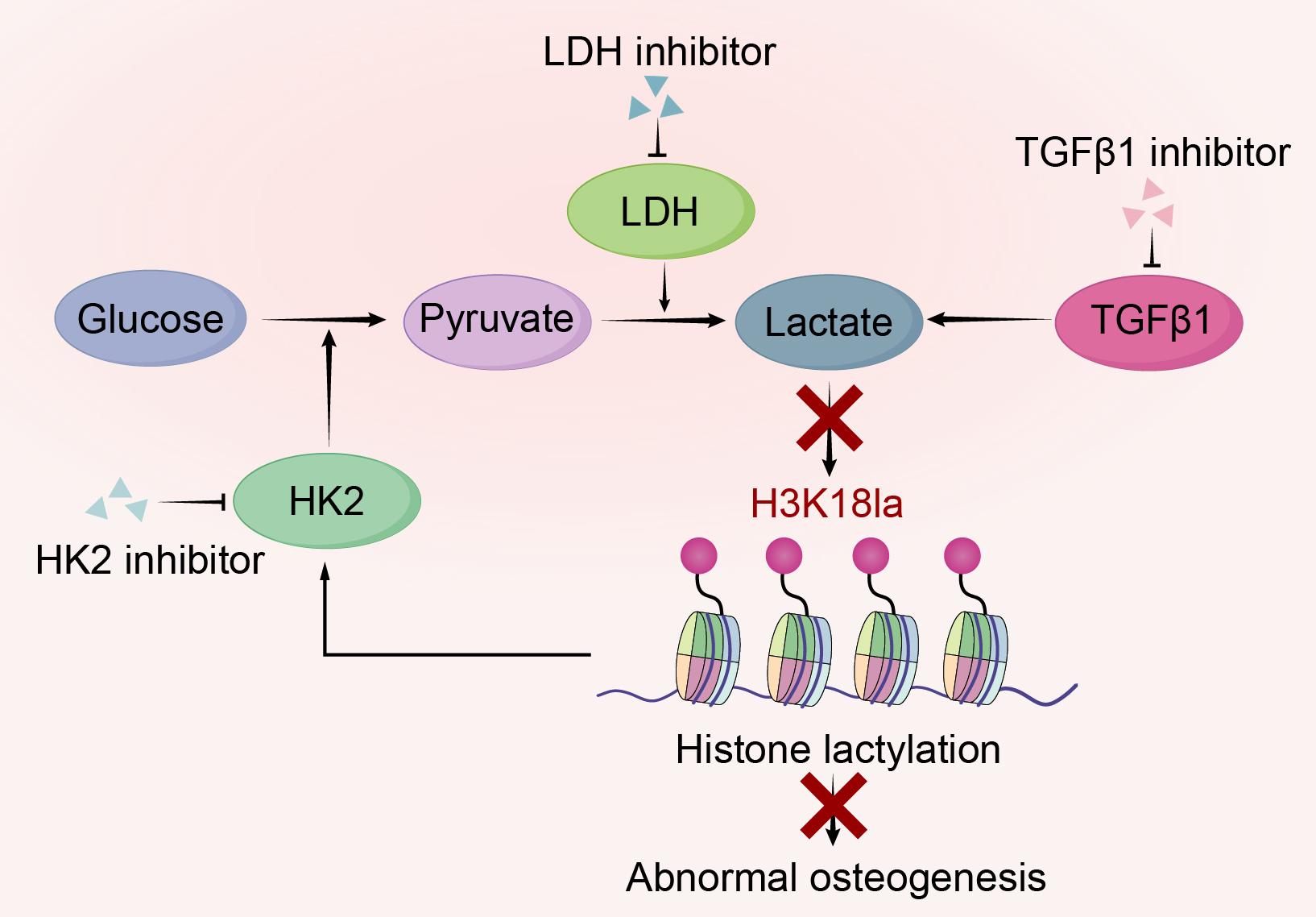

Supplement: Supplementary file 1 — Supplementary material 1. Figure S1: Characterization of primary ASPCs. A: The expression of the ASPCs surface markers CD73, CD90, and PDGFRA, the haematopoietic marker CD45 and the immune marker HLA-DR in isolated ASPCs at passage 1 was detected by flow cytometry. Figure S2: Oxamate and Nala had no effect on adipogenesis. A: The mature adipocytes with lipid droplets were visualized by Oil Red O staining on day 8 after 2-DG treatment. B: The expression of adipogenic marker genes PPAR γ, C/EBP α, and FABP4 was determined by Western blot in 2-DG treated FIL-ASPCs on day 3 of adipogenic differentiation. C: The mature adipocytes with lipid droplets were visualized by Oil Red O staining on day 8 after oxamate treatment. D: The expression of adipogenic marker genes PPAR γ, C/EBP α, and FABP4 was determined by Western blot in oxamate treated FIL-ASPCs on day 3 of adipogenic differentiation. E: The mature adipocytes with lipid droplets were visualized by Oil Red O staining on day 8 after Nala treatment. F: The expression of adipogenic marker genes PPAR γ, C/EBP α, and FABP4 was determined by Western blot in Nala treated FIL-ASPCs on day 3 of adipogenic differentiation. Full-length blots were presented in Figure S5. Figure S3: H3K18la activated the transcription of multiple genes related to osteogenesis. A: Genome browser tracks of CUT& Tag signal at the LEF1 and COL6A1 loci. B: Genome browser tracks of CUT& Tag signal at the ZBTB16 and FOXC1 loci. C: Genome browser tracks of CUT& Tag signal at the FGF18 and FBN1 loci. D: Genome browser tracks of CUT& Tag signal at the HAS2 and COL27A1 loci. Figure S4: Schematic diagram of the therapeutic strategies. Our research demonstrated that inhibiting HK2, LDH, and TGFβ1 may potentially suppress the abnormal osteogenic differentiation in FIL. Figure S5: Uncropped blot. [file 13287_2025_4651_MOESM1_ESM.zip › New folder/Figure S4.jpg]
