# Supplementary material for: H3K18 lactylation-hexokinase 2 positive feedback loop promotes osteogenesis of ASPCs in facial infiltrating lipomatosis
Source: Stem Cell Res Ther. 2025 Oct 1;16:538. doi: 10.1186/s13287-025-04651-5 (PMC12486737; doi:10.1186/s13287-025-04651-5)
Supplement: Supplementary file 3 — Supplementary material 3. [file 13287_2025_4651_MOESM3_ESM.docx]

**Table S2. Primers used in this study**

**Primer for qPCR:**

| **Gene/RNA** | **Forward primer (5'→3')** | **Reverse primer (5'→3')** |
| --- | --- | --- |
| GAPDH | CATCATCCCTGCCTCTACTGG | GTGGGTGTCGCTGTTGAAGTC |
| ALPL | CATACAGGATGGCAGTGAAGG | CCCGTGGCAACTCTATCTTTG |
| RUNX2 | CCGTCCATCCACTCTACCAC | ATGAAATGCTTGGGAACTGC |
| BMP7 | CCTCCATTGCTCGCCTTG | TATGCTGCTCATGTTTCCTAATAC |
| SP7 | TCCCTTTTCCCACTCATTC | GGGCAGACAGTCAGAAGAGC |
| BMP2 | GTGGACAAGACTCGGGATGAAA | ATCACGTAATGCCTGCTGTG |
| IBSP | GCATGCCTACTTTTATCCTCATTTAA | TCTTCTGAACTGTCATCTCCATTTTC |
| BGLAP | CACTCCTCGCCCTATTGGC | CCCTCCTGCTTGGACACAAAG |
| BMP4 | GATGTGGGCTGGAATGAC | GGTTGGTTGAGTTGAGGTG |
| HK2 | GAGCCACCACTCACCCTACT | CCAGGCATTCGGCAATGTG |
